# The efficacy and safety of tenecteplase versus alteplase for acute ischemic stroke: an updated systematic review, pairwise, and network meta-analysis of randomized controlled trials

**DOI:** 10.1007/s11239-022-02730-5

**Published:** 2022-11-30

**Authors:** Mohamed Abuelazm, Amith Reddy Seri, Ahmed K. Awad, Unaiza Ahmad, Abdelrahman Mahmoud, Ebraheem Albazee, Soumya Kambalapalli, Basel Abdelazeem

**Affiliations:** 1 Faculty of Medicine, Tanta University, Tanta, Egypt; 2 Department of Internal Medicine, McLaren Health Care, Flint, MI USA; 3 Department of Internal Medicine, Michigan State University, East Lansing, MI USA; 4 Faculty of Medicine, Ain-Shams University, Cairo, Egypt; 5 Punjab Medical College, Faisalabad, Pakistan; 6 Faculty of Medicine, Minia University, Minia, Egypt; 7 Kuwait Institute for Medical Specializations (KIMS), Kuwait City, Kuwait

**Keywords:** Tenecteplase, Alteplase, Stroke, TNK, AIS, Acute ischemic stroke, Systematic review, Meta-analysis, Clinical trials

## Abstract

Tenecteplase (TNK) is a promising candidate to replace alteplase as the standard of care for acute ischemic stroke (AIS); however, the optimal dosage is still to be investigated. Therefore, we aim to evaluate the safety and efficacy of TNK versus alteplase and to investigate the optimal TNK dosage. A systematic review, pairwise, and network meta-analysis synthesizing randomized controlled trials (RCTs) from WOS, SCOPUS, EMBASE, and PubMed until July 26^th^, 2022. We used the risk ratio (RR) for dichotomous outcomes presented with the corresponding 95% confidence interval (CI). We registered our protocol in PROSPERO with ID: CRD42022352038. Nine RCTs with a total of 3,707 patients were included. TNK significantly led to complete recanalization (RR: 1.27 with 95% CI [1.02, 1.57], P = 0.03); however, we found no difference regarding early neurological improvement (RR: 1.07 with 95% CI [0.94, 1.21], P = 0.33) and excellent neurological recovery (RR: 1.03 with 95% CI [0.96, 1.10], P = 0.42). Also, TNK was similar to alteplase regarding mortality (RR: 0.99 with 95% CI [0.82, 1.18], P = 0.88), intracranial haemorrhage (RR: 1.00 with 95% CI [0.85, 1.18], P = 0.99), and parenchymal hematoma (RR: 1.13 with 95% CI [0.83, 1.54], P = 0.44). TNK in the dose of 0.25 mg is a viable candidate to displace alteplase as the standard of care in patients with an AIS within 4.5 h of presentation due to its better rate of early neurological recovery and non-inferiority in terms of safety outcomes. However, the evidence regarding TNK’s role in AIS presenting after 4.5 h from symptoms onset, wake-up stroke, and minor stroke/TIA is still lacking, necessitating further double-blinded pragmatic RCTs in this regard.

**Supplementary Information:**

The online version contains supplementary material available at 10.1007/s11239-022-02730-5.

## Highlights

- We conducted a systematic review and network meta-analysis to investigate the efficacy and safety of tenecteplase versus alteplase for thrombolysis in patients with acute ischemic stroke and to investigate the most effective tenecteplase dosage.
- Given its higher rate of early neurological recovery and non-inferiority in terms of safety outcomes, tenecteplase in the dose of 0.25 mg is a strong candidate to replace alteplase as the standard of care in patients with acute ischemic stroke who present within 4.5 hours of symptoms onset.
- Tenecteplase's potential in acute ischemic stroke presenting after 4.5 hours from the onset of symptoms, wakeup stroke, and mild stroke/TIA is still inconclusive.

## Introduction

Worldwide, stroke still ranks as the second-leading cause of death and the third-leading cause of composite death and disability (as expressed by disability-adjusted life-years lost—DALYs), amounting to a global health expenditure of over 721 billion US$ (0.66% of the global GDP) [1]. Annually, approximately 700,000 people in the United States experience an acute ischemic stroke (AIS) [2], constituting 85% of stroke cases. There have been greater advances in terms of the management of AIS in comparison with hemorrhagic stroke. Novel treatments such as IV thrombolysis (IVT), and more recently, mechanical thrombectomy (MT) for large vessel occlusion (LVO) have reduced mortality by ten percent compared with the older treatments and improved long-term disability prevention rates after AIS [3, 4].

AIS management guidelines in Europe [5], Canada [6], the United States [7], and the United Kingdom [8], recommend intravenous thrombolysis with the tissue plasminogen activator alteplase (t-PA) within 4.5 h after the onset of stroke and MT within 24 h after onset. Alteplase is the only thrombolytic drug that is FDA-approved for thrombolysis in AIS. Alteplase can lead to a 28% decrease in disability at 90 days and rapid symptom improvement when given within the 4.5 h window period [9]. Despite being promising for a disability-free recovery, implementation of alteplase is restricted due to the narrow time window and adverse effects of alteplase, such as a 6% risk of symptomatic hemorrhage [10]. Moreover, alteplase has demonstrated limited fibrinolytic efficacy; achieving arterial recanalization in fewer than 50% of patients [11]. Also, In patients who achieve recanalization, only 50% recanalize within two hours after drug administration [12]. Furthermore, there has been a rising concern over alteplase’s negative effects on the ischaemic brain, including cytotoxicity and increased permeability of the blood–brain–barrier facilitating cerebral edema [13–16].

In this scenario, a thrombolytic that is safe, easy to administer, and effective can broaden the acceptance of thrombolytic therapy for stroke. Tenecteplase (TNK), a genetically modified variant of alteplase, has been approved by the FDA for thrombolysis in acute myocardial infarction since 2000 after reports from the ASSENT 2 Trial [17]. Multiple clinical trials comparing TNK with alteplase in acute MI have shown that TNK induces faster coronary reperfusion with similar mortality rates [18]. Success in acute MI treatment and animal models for AIS has prompted interest in the replacement of alteplase for TNK therapy in AIS. TNK has several advantages that make it an appealing alternative; it is generally cost-effective, has a high fibrin specificity and longer plasma half-life, enhanced plasminogen Activator Inhibitor-1 (PAI-1) resistance, and can be dispensed as a single bolus; allowing swift treatment without the need for additional equipment such as infusion pumps, making it applicable in the pre-hospital settings [19].

A previous systematic review and network meta-analysis concluded that TNK is at least safe and effective as an alteplase for AIS [20]. However, multiple randomized controlled trials (RCTs) have been recently published with a significantly larger number of participants and conclusions favoring TNK over alteplase [19, 21–23]. Furthermore, in the absence of generalizable results owing to heterogeneous patient population traits, variability in doses administered, and differing clinical endpoints and outcomes evaluated; the relative superiority of TNK over alteplase remains controversial. Therefore, we aim to update the synthesized evidence on the efficacy and safety of TNK versus alteplase for thrombolysis in patients with AIS and to investigate the most effective dosage of TNK.

## Methodology

### Protocol registration

This systematic review network meta-analysis was performed according to the Preferred Reporting Items for Systematic Reviews and Meta-Analysis extension statement for network meta-analyses [24] and the Cochrane Handbook of Systematic reviews and meta-analysis [25]. The review protocol was published in the International prospective register of systematic reviews (PROSPERO) with ID: CRD42022352038.

### Data sources & search strategy

Two reviewers (B.A. and M.A.) independently conducted an electronic systematic search on PubMed (MEDLINE), EMBASE, Web of Science, SCOPUS, and Cochrane Central Register of Controlled Trials (CENTRAL) until July 26th, 2022, without using any search filters. The search strategy for each database is illustrated in (Table S1).

### Eligibility criteria

We included RCTs with the following PICO criteria: population (P): adult patients presenting with AIS and undergoing thrombolysis; intervention (I): TNK irrespective of the dose; control (C): alteplase; outcomes (O): efficacy outcomes: early neurological improvement measured by ≥ 4 points reduction in the National Institutes of Health Stroke Scale (NIHSS), excellent neurological recovery (modified Rankin Scale (mRS) 0–1), good neurological recovery (mRS 0–2), and successful reperfusion measured by modified treatment in cerebral ischemia classification or Thrombolysis in Cerebral Infarction (TICI). Furthermore, safety outcomes; all-cause mortality, poor neurological recovery (mRS 4–6), any intracranial hemorrhage (ICH), symptomatic ICH, and any parenchymal hematoma. Conference abstracts, posters, letters to editor, non-randomized trials, single-arm trials, and observational studies were excluded.

### Selection process

The selection process was conducted over two steps, first, four reviewers (A.R.S., A.M., E.A., and K.S.) independently screened the titles and abstracts of the retrieved records using Covidence online software [26]. Then they independently screened the full-texts confirming eligibility using the previous eligibility criteria. Disagreements were resolved by discussion or inviting (B.A.) to reach a consensus.

### Data extraction

Using a standardized extraction sheet, four reviewers (A.R.S., A.M., E.A., and K.S.) independently extracted the following data from the eligible trials: study characteristics (first author name, year of publication, country, study design, total participants, recruitment duration, intervention dosages, main inclusion criteria, and time window); baseline information (age, sex, number of patients in each arm, onset to infusion time, and stroke risk factors); efficacy outcomes data; and safety outcomes data. Disagreements were resolved through discussion.

### Risk of bias and quality assessment

Four reviewers (A.R.S., A.M., E.A., and K.S.) independently investigated the quality of the included trials following The Cochrane Collaboration's tool for assessing the risk of bias (ROB) in randomized trials [27], based on the following domains: random sequence generation (selection bias), allocation concealment (selection bias), blinding of participants and personnel (performance bias), blinding of outcome assessment (detection bias), incomplete outcome data (attrition bias), selective reporting (reporting bias), and other potential sources of bias. Disagreements were resolved by discussion. Two reviewers (M.T. and B.A.), guided by the Grading of Recommendations Assessment, Development, and Evaluation (GRADE) guidelines [28], appraised the quality of the outcome findings. Imprecision, indirectness, inconsistency, publication bias, and risk of bias were considered. Our results about the quality of evidence were rationalized, clarified, and included for each outcome. Any discrepancies were handled through discussion.

### Statistical analysis

For the pairwise meta-analysis, we used Revman version 5.4 [29] to pool dichotomous outcomes using risk ratio (RR) along with the corresponding 95% confidence interval (CI). We used the fixed-effect model; however, the random-effect model was used in case of significant heterogeneity. Statistical heterogeneity was evaluated by calculating I2 and conducting a chi-squared test. P-value 0.05 was considered significant, and I2 > 50% indicated substantial heterogeneity, in which case sensitivity analysis was performed by removing one study at a time to determine if there is one study that affects the overall effect estimate.

For network meta-analysis, we performed a network meta-analysis using a frequentist framework [24], pooling dichotomous outcomes using risk ratio (RR) along with the corresponding 95% confidence interval (CI). Analysis was performed using the R-software netmeta and netrank package (R version 4.2.0) and meta-insight software [30–32]. Finally, because we only included less than ten studies in each outcome, we did not conduct funnel plots to reveal publication bias, as advised by Egger et al. [33].

## Results

### Search results and study selection

**Fig. 1:**
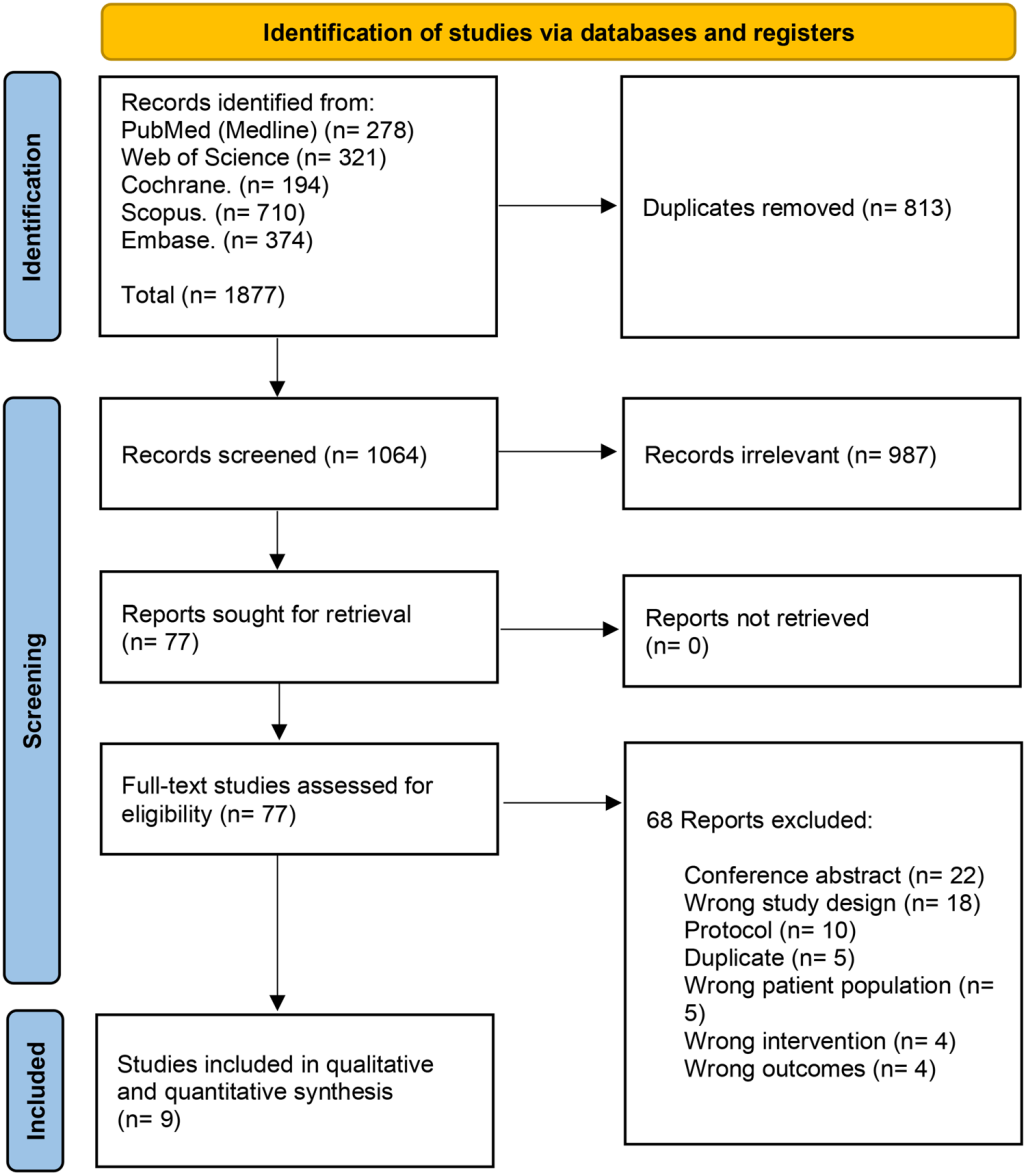
PRISMA flow chart of the screening process

We imported 1877 records after searching databases. Eight hundred and thirteen duplicates were removed using Covidence, leaving 1064 records for the title and abstract screening. We excluded 987 irrelevant records and screened 77 full-text articles, and finally included nine RCTs [19, 21–23, 34–38] (Fig. 1).

### Characteristics of included studies

Nine RCTs met our inclusion criteria [19, 21–23, 34–38] with a total of 3,707 patients included; of these: 1,967 were allocated to TNK (intervention), and 1,740 were allocated to alteplase (control). Seven trials were multicenter [21–23, 35–38], whereas only two RCTs were single-center trials [19, 34]. The time window was 4.5 h in six trials [19, 21, 22, 34, 35, 37], three hours in two trials [23, 36], and less than six hours in one trial [38]. Table 1 and Table 2 demonstrate the summary and baseline characteristics of the included studies, respectively.

**Table 1:** Summary of the included studies

Study ID	Study Design	Country	Recruitment duration	Total sample size, N	Dosages (mg/kg)		Timing after symptoms onset
					Tenecteplase	Alteplase	
Bivard et al. 2022 [19]	Phase 2, single center, PROBE	Australia	From June 2019 to November 2021	N = 104	(0·25 mg/kg)	(0·9 mg/kg)	4.5 h
Campbell et al. 2018 [35]	Phase 2, multicenter, PROBE	Australia and New Zealand	From March 2015 to October 2017	N = 202	(0·25 mg/kg)	(0·9 mg/kg)	4.5 h
Haley et al. 2010 [36]	Phase 2B/3, multicenter, double-blinded, prematurely terminated RCT	USA	From March 2006 to December 2008	N = 112	Group 1 = 0.1 mg/kg Group 2 = 0.25 mg/kg Group 3 = 0.4 mg/kg	(0.9 mg/kg)	3 h
Huang et al. 2015 [34]	Phase 2, single center, PROBE	Scotland	From January 2012 to September 2013	N = 96	(0·25 mg/kg)	(0·9 mg/kg)	4.5 h
Kvistad et al. 2022 [21]	Phase 3, multicenter, PROBE	Norway	From October 2019 to September 2021	N = 204	(0·4 mg/kg) [maximum 40 mg]	(0·9 mg/kg)	4.5 h
Li et al. 2021 [23]	Phase 2, multicenter, PROBE	China	From May 2018 to February 2020	N = 236	Group 1 = 0.1 mg/kg Group 2 = 0.25 mg/kg Group 3 = 0.32 mg/kg	(0·9 mg/kg)	3 h
Logallo et al. 2017 [37]	Phase 3, multicenter, PROBE	Norway	From September 2012 to September 2016	N = 1,100	(0·4 mg/kg)	(0·9 mg/kg)	4.5 h
Menon et al. 2022 [22]	Phase 3, multicenter, PROBE	Canada	From December 2019 to January 2022	N = 1,577	(0·25 mg/kg)	(0·9 mg/kg)	4.5 h
Parsons et al. 2012 [38]	Phase 2B, multicenter, PROBE	Australia	From 2008 to 2011	N = 75	Group 1 = 0.1 mg/kg Group 2 = 0.25 mg/kg	(0·9 mg/kg)	less than 6 h

*PROBE* prospective, randomized, open-label, blinded outcome study, *RCT* randomized controlled trial, *N* number, *mg* milligram, *kg* kilogram

**Table 2:** Baseline characteristics of the included studies

Study ID	Sample size, n		Age (years)		Male, n(%)		Onset to treatment time, (min)		Baseline NIHSS, mean (SD)	
	Tenecteplase	Alteplase	Tenecteplase	Alteplase	Tenecteplase	Alteplase	Tenecteplase	Alteplase	Tenecteplase	Alteplase
Bivard et al. 2022 [19]	n = 55	n = 49	73.33 ± 18.27	71.33 ± 14.51	33 (60%)	30 (61%)	107.33 ± 67.75	96.33 ± 49.64	9 ± 6.85	10 ± 9.16
Campbell et al. 2018 [35]	n = 101	n = 101	70.4 ± 15.1	71.9 ± 13.7	58 (57%)	52 (51%)	127.6 ± 40.61	138 ± 54.15	17 ± 7.52	17 ± 7.52
Haley et al. 2010 [36]	TNK 0.1: n = 31 TNK 0.25: n = 31 TNK 0.4: n = 19	n = 31	TNK 0.1: 67 ± 19 TNK 0.25: 69 ± 15 TNK 0.4: 68 ± 16	72 ± 16	TNK 0.1: 12 (39%) TNK 0.25: 16 (52%) TNK 0.4: 13 (68%)	17 (51%)	NA	NA	TNK 0.1: 8 ± 4.66 TNK 0.25: 10.33 ± 7 TNK 0.4: 10.33 ± 9.6	11.6 ± 9.32
Huang et al. 2015 [34]	n = 47	n = 49	71 ± 13	71 ± 12	30 (64%)	31 (63%)	184 ± 44	192 ± 45	13 ± 6.88	11.6 ± 6.11
Kvistad et al. 2022 [21]	n = 100	n = 104	73·2 ± 12·6	68·6 ± 15·6	45 (45%)	53 (51%)	103.16 ± 51.90	105 ± 52.62	13.4 ± 6.6	13.2 ± 6.4
Li et al. 2021 [23]	TNK 0.1: n = 60 TNK 0.25: n = 57 TNK 0.32: n = 60	n = 59	TNK 0.1: 62.4 ± 11.1 TNK 0.25: 64.3 ± 12.8 TNK 0.32: 64.8 ± 12.1	66.5 ± 12.6	TNK 0.1: 48 (80%) TNK 0.25: 42 (73.7%) TNK 0.32: 42 (70%)	38 (64.4%)	TNK 0.1: 135 ± 105.6 TNK 0.25: 136 ± 75.3 TNK 0.32:145.33 ± 114.7	119.3 ± 128.42	TNK 0.1: 7.33 ± 3.80 TNK 0.25: 8.33 ± 5.33 TNK 0.32: 8.5 ± 4.6	8.33 ± 5.32
Logallo et al. 2017 [37]	n = 549	n = 551	70.8 ± 14.4	71.2 ± 13.2	321 (58%)	339 (62%)	125.67 ± 75	121.6 ± 69.87	5.6 ± 5.4	5.8 ± 5.2
Menon et al. 2022 [22]	n = 806	n = 771	73.33 ± 14.85	72.67 ± 15.6	424 (52.6%)	398 (51.6%)	135 (69.05%)	138 ± 69.1	10.33 ± 7.43	11 ± 8.14
Parsons et al. 2012 [38]	TNK 0.1: n = 25 TNK 0.25: n = 25	n = 25	TNK 0.1: 72 ± 6.9 TNK 0.25: 68 ± 9.4	70 ± 8.4	TNK 0.1: 13 (52%) TNK 0.25: 13 (52%)	12 (48%)	TNK 0.1: 3.1 ± 0.9 (h) TNK 0.25: 3.0 ± 0.7 (h)	2.7 ± 0.8 (h)	TNK 0.1: 14.5 ± 2.3 TNK 0.25: 14.6 ± 2.3	14 ± 2.3

Study ID	Comorbidities, n(%)									
	AF		HTN		DM		Dyslipidaemia		Smoking	
	Tenecteplase	Alteplase	Tenecteplase	Alteplase	Tenecteplase	Alteplase	Tenecteplase	Alteplase	Tenecteplase	Alteplase
Bivard et al. 2022 [19]	8 (15%)	7 (15%)	30 (55%)	31 (63%)	11 (30%)	17 (35%)	21 (38%)	22 (45%)	8 (15%)	9 (18%)
Campbell et al. 2018 [35]	27 (27%)	40 (40%)	64 (63%)	63 (62%)	10 (10%)	18 (18%)	N/A	N/A	18 (18%)	11 (11%)
Haley et al. 2010 [36]	NA	NA	TNK 0.1: 25 (81%) TNK 0.25: 25 (81%) TNK 0.4: 17 (90%)	22 (71%)	TNK 0.1: 6 (19%) TNK 0.25: 7 (23%) TNK 0.4: 4 (21%)	4 (13%)	TNK 0.1: 16 (52%) TNK 0.25: 15 (48%) TNK 0.4: 8 (42%)	17 (55%)	TNK 0.1: 2 (6.5%) TNK 0.25: 7 (23%) TNK 0.4: 0 (0%)	7 (23%)
Huang et al. 2015 [34]	19 (40%)	15 (31%)	20 (43%)	28 (57%)	7 (15%)	7 (14%)	4 (9%)	7 (14%)	13 (28%)	10 (20%)
Kvistad et al. 2022 [21]	9 (9%)	8 (8%)	56 (56%)	48 (46%)	17 (17%)	11 (11%)	30 (30%)	33 (32%)	24 (24%)	25 (24%)
Li et al. 2021 [23]	TNK 0.1: 8 (13.3%) TNK 0.25: 4 (7.0%) TNK 0.32: 14 (23.3%)	6 (10.2%)	TNK 0.1: 43 (71.7%) TNK 0.25: 37 (64.9%) TNK 0.32: 35 (58.3%)	42 (71.2%)	TNK 0.1: 14 (23.3%) TNK 0.25: 9 (15.8%) TNK 0.32: 15 (25%)	11 (18.6%)	TNK 0.1: 17 (28.3%) TNK 0.25: 13 (22.8%) TNK 0.32: 10 (16.7%)	11 (18.6%)	TNK 0.1: 25 (41.7%) TNK 0.25: 25 (43.9%) TNK 0.32: 21 (35%)	24 (40.7%)
Logallo et al. 2017 [37]	50 (9%)	69 (13%)	246 (45%)	236 (43%)	72 (13%)	74 (13%)	61 (11%)	65 (12%)	169 (31%)	177 (32%)
Menon et al. 2022 [22]	NA	NA	NA	NA	NA	NA	NA	NA	NA	NA
Parsons et al. 2012 [38]	TNK 0.1: 9 (36%) TNK 0.25: 13 (52%)	6 (24%)	TNK 0.1: 16 (64%) TNK 0.25: 16 (64%)	15 (60)%	TNK 0.1: 8 (32%) TNK 0.25: 6 (24%)	1 (4%)	TNK 0.1: 13 (52%) TNK 0.25: 15 (60%)	9 (36%)	TNK 0.1: 9 (36%) TNK 0.25: 5 (20%)	1 (4%)

*NIHSS* national institute of health stroke scale, *AF* atrial fibrillation, *HTN* hypertension, *DM* diabetes mellitus, *NA* not available, *n* number, *SD* standard deviation

### Risk of bias and quality of evidence

**Fig. 2:**
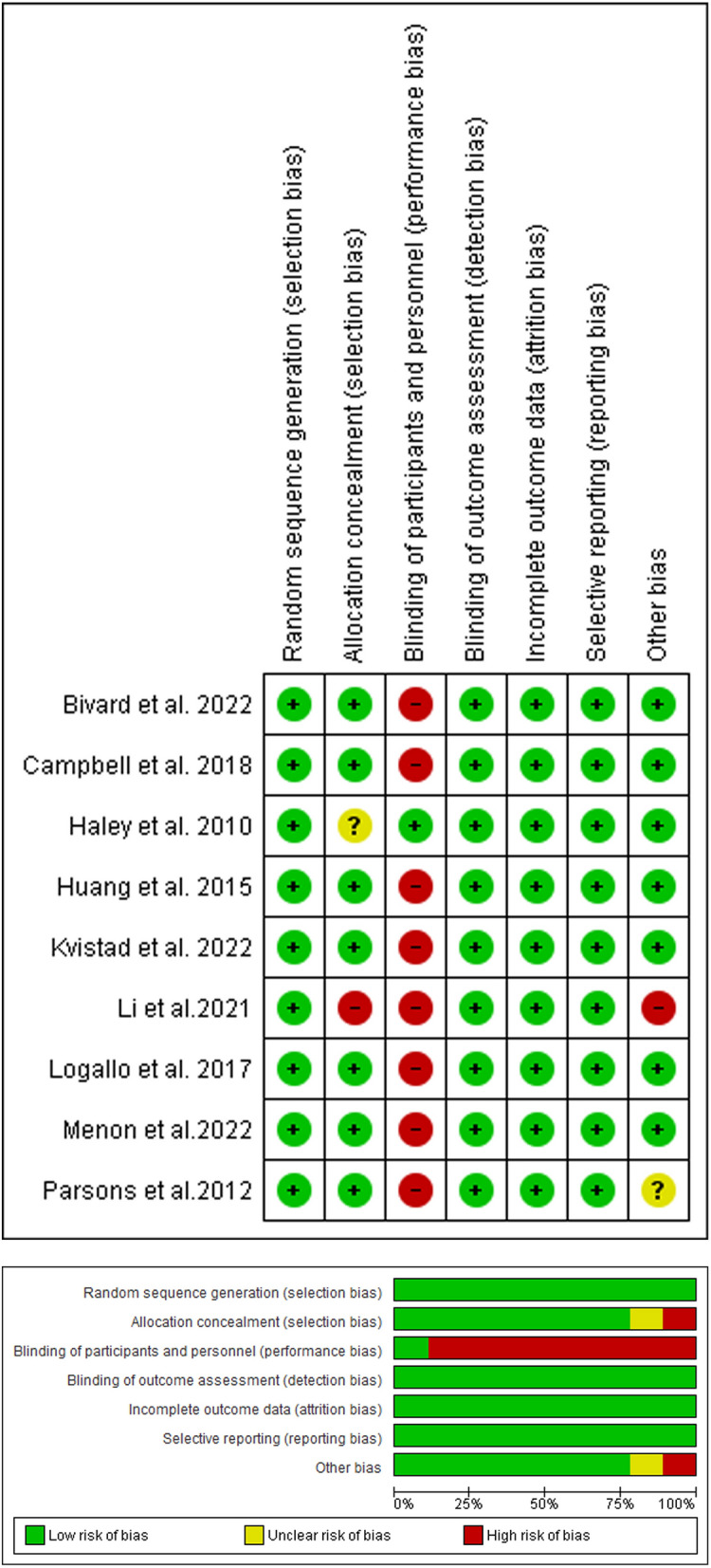
Quality assessment of risk of bias in the studies in the meta-analysis. The upper panel presents a schematic representation of risks (low = red, unclear = yellow, and high = red) for specific types of biases of each of the studies in the review. The lower panel presents risks (low = red, unclear = yellow, and high = red) for the subtypes of biases of the combination of studies included in this review

We assessed the quality of the included studies according to the Cochrane risk of bias tool as shown in (Fig. 2). All the included trials had a low risk of random sequence generation bias. All the included studies had a low risk of allocation concealment bias except Haley et al. 2010 [36], which had an unclear risk, while Li et al. 2021 [23] had a high risk of bias. Moreover, all included trials had a high risk of performance bias except for Haley et al. 2010 [36], which had a low risk of performance bias. Furthermore, all included trials had a low risk of detection bias. For the attrition and reporting bias, all our included studies had a low risk of bias. Finally, all the included studies had a low risk of other bias except for Li et al. 2021[23], which had a high risk of bias, and Parsons et al. 2012 [38], which had an unclear risk of bias. Author judgments are furtherly clarified in (Table S2). Finally, the quality of evidence is illustrated in (Table S3).

### Efficacy outcomes

#### Early neurological improvement

**Fig. 3:**
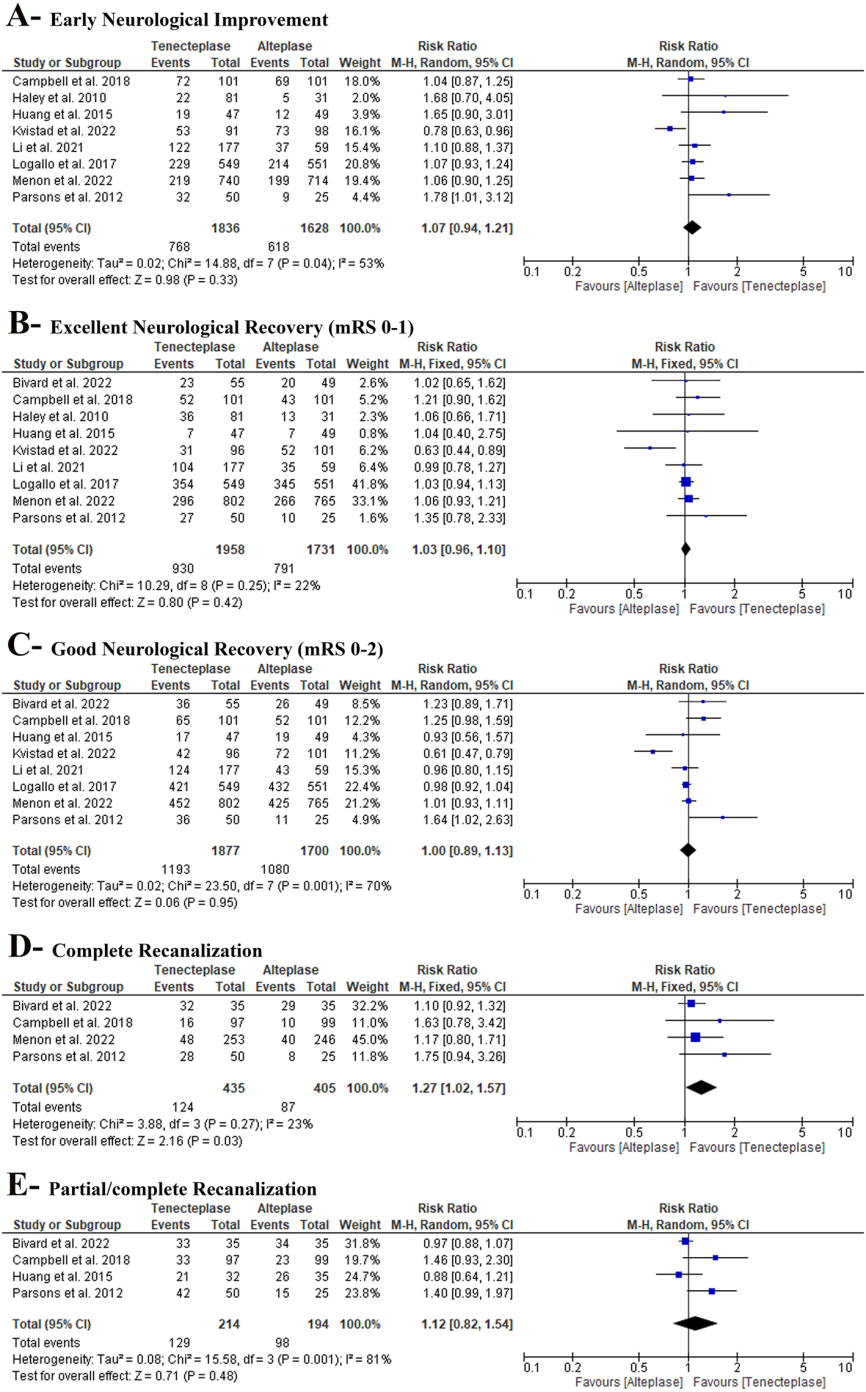
Forest plot of the efficacy outcomes (**A** early neurological improvement, **B**- excellent neurological recovery, **C**- good neurological recovery, **D**- complete recanalization, and **E**- partial/complete recanalization), *RR* risk ratio, *CI* confidence interval

In the pairwise meta-analysis, we found no difference between TNK and alteplase (RR: 1.07 with 95% CI [0.94, 1.21], P = 0.33) (low-quality evidence) (Fig. 3-A, Table S3). Pooled studies were heterogenous (P = 0.04, I^2^ = 53%). Heterogeneity was best resolved after excluding Kvistad et al. [21] (P = 0.41, I^2^ = 1%) (Table S4). After excluding Kvistad et al. [21], pooled risk ratio favored TNK (RR: 1.09 with 95% CI [1.01, 1.19], P = 0.04) (Table S4).

In network meta-analysis, all TNK doses showed no statistically significant difference, except TNK-0.25, which showed a statistically significant higher risk for early neurological improvement (RR: 1.24 with 95% CI [1.02, 1.49]) (Table 3, Figures S1-A, S2, S3). No heterogeneity was observed (I^2^ = 0%).

**Table 3:** Ranking table for all our network meta-analyses’ outcomes

Early neurological improvement				
TNK 0.4				
0.93 [0.72; 1.19]	Alteplase			
0.84 [0.54; 1.29]	0.90 [0.63; 1.29]	TNK 0.32		
0.80 [0.56; 1.16]	0.87 [0.66; 1.14]	0.96 [0.66; 1.39]	TNK 0.1	
0.75 [0.55; 1.02]	0.81 [0.67; 0.98]	0.90 [0.63; 1.27]	0.93 [0.72; 1.20]	TNK 0.25
Excellent neurological recovery (mRS 0–1)				
TNK 0.4				
0.97 [0.70; 1.34]	TNK 0.1			
0.90 [0.73; 1.10]	0.93 [0.71; 1.21]	Alteplase		
0.86 [0.59; 1.26]	0.89 [0.62; 1.26]	0.96 [0.69; 1.33]	TNK 0.32	
0.79 [0.61; 1.02]	0.81 [0.63; 1.06]	0.88 [0.75; 1.03]	0.92 [0.66; 1.27]	TNK 0.25
Good neurological recovery (mRS 0–2)				
TNK 0.4				
0.83 [0.55; 1.26]	TNK 0.1			
0.82 [0.52; 1.29]	0.98 [0.66; 1.46]	TNK 0.32		
0.81 [0.62; 1.07]	0.98 [0.72; 1.33]	0.99 [0.69; 1.43]	Alteplase	
0.71 [0.51; 0.98]	0.85 [0.63; 1.14]	0.86 [0.60; 1.24]	0.87 [0.72; 1.04]	TNK 0.25
Poor neurological recovery (mRS 4–6)				
TNK 0.4				
0.96 [0.59; 1.58]	TNK 0.1			
0.86 [0.68; 1.10]	0.90 [0.56; 1.46]	Alteplase		
0.83 [0.43; 1.63]	0.87 [0.44; 1.72]	0.97 [0.50; 1.87]	TNK 0.32	
0.69 [0.45; 1.07]	0.72 [0.40; 1.30]	0.80 [0.55; 1.17]	0.83 [0.39; 1.76]	TNK 0.4
Partial/complete recanalization				
TNK 0.4				
0.97 [0.58; 1.61]	Alteplase			
0.84 [0.52; 1.37]	0.87 [0.67; 1.14]	TNK 0.25		
Complete recanalization				
TNK 0.1				
0.64 [0.29; 1.40]	Alteplase			
0.47 [0.22; 0.99]	0.73 [0.53; 1.01]	TNK 0.25		
All-cause mortality at 90 days				
TNK 0.25				
1.04 [0.45; 2.40]	TNK 0.1			
0.99 [0.30; 3.30]	0.96 [0.29; 3.12]	TNK 0.32		
0.87 [0.59; 1.29]	0.84 [0.38; 1.85]	0.88 [0.27; 2.80]	Alteplase	
0.66 [0.34; 1.29]	0.64 [0.24; 1.66]	0.67 [0.18; 2.41]	0.76 [0.43; 1.35]	TNK 0.4
Any intracranial hemorrhage				
TNK 0.25				
0.84 [0.53; 1.34]	Alteplase			
0.72 [0.29; 1.81]	0.85 [0.35; 2.10]	TNK 0.1		
0.54 [0.17; 1.71]	0.64 [0.21; 2.01]	0.75 [0.25; 2.30]	TNK 0.32	
0.55 [0.28; 1.07]	0.65 [0.38; 1.11]	0.76 [0.28; 2.04]	1.00 [0.29; 3.43]	TNK 0.4
Symptomatic intracranial hemorrhage				
TNK 0.25				
0.95 [0.60; 1.50]	Alteplase			
0.99 [0.28; 3.46]	1.04 [0.31; 3.50]	TNK 0.1		
0.93 [0.17; 5.05]	0.98 [0.19; 5.15]	0.94 [0.20; 4.32]	TNK 0.32	
0.57 [0.28; 1.19]	0.60 [0.33; 1.11]	0.58 [0.15; 2.19]	0.62 [0.11; 3.56]	TNK 0.4
Any parenchymal hematoma				
TNK 0.1				
0.66 [0.11; 4.08]	TNK 0.25			
0.53 [0.09; 2.97]	0.80 [0.39; 1.64]	Alteplase		
0.07 [0.01; 0.85]	0.11 [0.02; 0.73]	0.14 [0.03; 0.79]	TNK 0.4	

*TNK* Tenecteplase, all data are reported in risk ratio (RR) and 95% confidence interval (CI)

#### Excellent neurological recovery (mRS 0–1).

In pairwise meta-analysis, we found no difference between TNK and alteplase (RR: 1.03 with 95% CI [0.96, 1.10], P = 0.42) (low-quality evidence) (Fig. 3-B, Table S3). Pooled studies were homogenous (P = 0.25, I^2^ = 22%).

In network meta-analysis, all TNK doses showed no statistically significant difference, compared to alteplase: TNK 0.1 (RR: 0.93 with 95% CI [0.71, 1.21]), TNK 0.25 (RR: 1.14 with 95% CI [0.97, 1.33]), TNK 0.32 (RR: 1.05 with 95% CI [0.75, 1.45]), and TNK 0.4 (RR: 0.9 with 95% CI [0.73, 1.10]) (Table 3, Figures S1-B, S4, S5). No significant heterogeneity was observed (I^2^ = 12%).

#### Good neurological recovery (mRS 0–2).

In the pairwise meta-analysis, we found no difference between TNK and alteplase (RR: 1.00 with 95% CI [0.89, 1.13], P = 0.95) (very low-quality evidence) (Fig. 3-C, Table S3). Pooled studies were heterogenous (P = 0.001, I^2^ = 70%). Heterogeneity was best resolved after excluding Kvistad et al. [21] (P = 0.12, I^2^ = 41%); however, after excluding Kvistad et al. [21], there was no difference between TNK and alteplase (RR: 1.04 with 95% CI [0.95, 1.13], P = 0.39) (Table S4).

In network meta-analysis, all TNK doses showed no statistically significant difference, compared to alteplase: TNK 0.1 (RR: 0.98 with 95% CI [0.72, 1.33]), TNK 0.25 (RR: 1.15 with 95% CI [0.96, 1.38]), TNK 0.32 (RR: 0.99 with 95% CI [0.69, 1.43]), and TNK 0.4 (RR: 0.81 with 95% CI [0.62, 1.07]) (Table 3, Figures S1-C, S6, S7). No heterogeneity was observed (I^2^ = 0%).

#### Complete recanalization

In pairwise meta-analysis, pooled risk ratio favored TNK (RR: 1.27 with 95% CI [1.02, 1.57], P = 0.03) (low-quality evidence) (Fig. 3-D, Table S3). Pooled studies were homogenous (P = 0.27, I^2^ = 23%).

In network meta-analysis, TNK 0.1, and TNK 0.25 showed no statistically significant difference (RR: 0.64 with 95% CI [0.29, 1.40]), and (RR: 1.37 with 95% CI [0.99, 1.89]), respectively (Table 3, Figures S1-D, S8, S9). No significant heterogeneity was observed (I^2^ = 24%).

#### Partial/complete recanalization

In the pairwise meta-analysis, we found no difference between TNK and alteplase (RR: 1.12 with 95% CI [0.82, 1.54], P = 0.48) (very low-quality evidence) (Fig. 3-E, Table S3). Pooled studies were heterogenous (P = 0.001, I^2^ = 81%). Heterogeneity was not resolved by sensitivity analysis (Table S4).

In network meta-analysis, TNK 0.1, and TNK 0.25 showed no statistically significant difference (RR: 0.97 with 95% CI [0.58, 1.61]), and (RR: 1.15 with 95% CI [0.88, 1.50]), respectively (Table 3, Figures S1-E, S10,S11). No significant heterogeneity was observed (I^2^ = 32%).

### Safety outcomes

#### Poor neurological recovery (mRS 4–6).

**Fig. 4:**
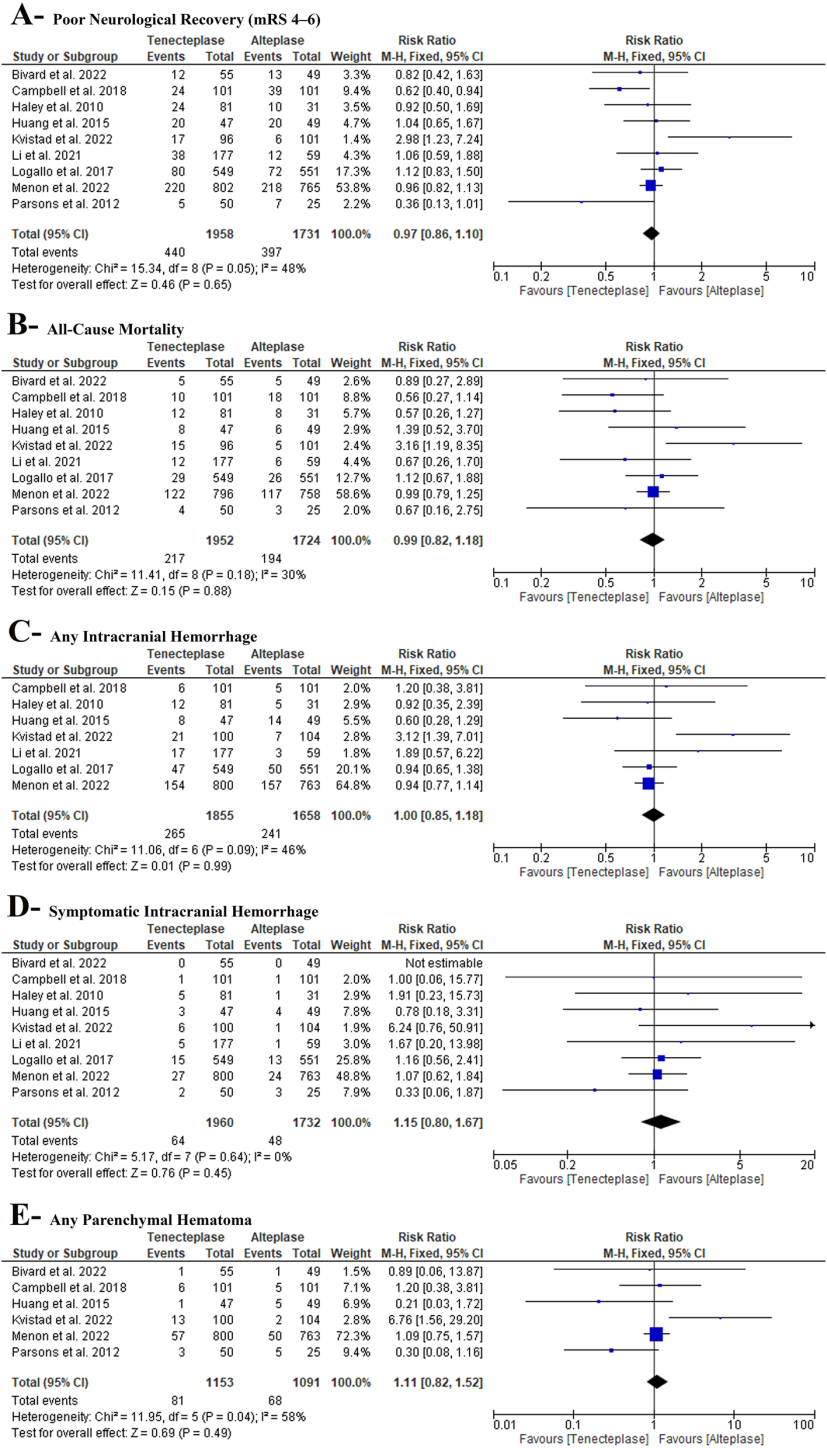
F or est plot of the safety outcomes (**A**- poor neurological improvement, **B**- all-cause mortality at 90 days, **C**- any intracranial hemorrhage, **D**- symptomatic intracranial hemorrhage, and **E**- any parenchymal hematoma), *RR* risk ratio, *CI* confidence interval

In the pairwise meta-analysis, we found no difference between TNK and alteplase (RR: 0.97 with 95% CI [0.86, 1.10], P = 0.65) (low-quality evidence) (Fig. 4-A, Table S3). Pooled studies were homogenous (P = 0.05, I^2^ = 48%).

In network meta-analysis, all TNK doses showed no statistically significant difference, compared to alteplase: TNK 0.1 (RR: 0.90 with 95% CI [0.56, 1.46]), TNK 0.25 (RR: 0.86 with 95% CI [0.68, 1.10]), TNK 0.32 (RR: 1.04 with 95% CI [0.53, 2.01]), and TNK 0.4 (RR: 1.25 with 95% CI [0.85, 1.82]) (Table 3, Figures S12-A, S13, S14). No heterogeneity was observed (I^2^ = 0%).

#### All-cause mortality at 90 days

In the pairwise meta-analysis, we found no difference between TNK and alteplase (RR: 0.99 with 95% CI [0.82, 1.18], P = 0.88) (high-quality evidence) (Fig. 4-B, Table S3). Pooled studies were homogenous (P = 0.18, I^2^ = 30%).

In network meta-analysis, all TNK doses showed no statistically significant difference, compared to alteplase: TNK 0.1 (RR: 0.84 with 95% CI [0.38, 1.85]), TNK 0.25 (RR: 0.87 with 95% CI [0.59, 1.29]), TNK 0.32 (RR: 0.88 with 95% CI [0.27, 2.80]), and TNK 0.4 (RR: 1.32 with 95% CI [0.74, 2.33]) (Table 3, Figures S12-B, S15, S16). No significant heterogeneity was observed (I^2^ = 12%).

#### Any ICH

In the pairwise meta-analysis, we found no difference between TNK and alteplase (RR: 1.00 with 95% CI [0.85, 1.18], P = 0.99) (moderate-quality evidence) (Fig. 4-C, Table S3). Pooled studies were homogenous (P = 0.09, I^2^ = 46%).

In network meta-analysis, all TNK doses showed no statistically significant difference, compared to alteplase: TNK 0.1 (RR: 1.17 with 95% CI [0.48, 2.88]), TNK 0.25 (RR: 0.84 with 95% CI [0.53, 1.34]), TNK 0.32 (RR: 1.55 with 95% CI [0.50, 4.85]), and TNK 0.4 (RR: 1.55 with 95% CI [0.90, 2.64]) (Table 3, Figures S12-C, S17, S18). No significant heterogeneity was observed (I^2^ = 12%).

#### Symptomatic ICH

In the pairwise meta-analysis, we found no difference between TNK and alteplase (RR: 1.15 with 95% CI [0.80, 1.67], P = 0.45) (low-quality evidence) (Fig. 4-D, Table S3). Pooled studies were homogenous (P = 0.64, I^2^ = 0%).

In network meta-analysis, all TNK doses showed no statistically significant difference, compared to alteplase: TNK 0.1 (RR: 0.96 with 95% CI [0.29, 3.24]), TNK 0.25 (RR: 0.95 with 95% CI [0.60, 1.50]), TNK 0.32 (RR: 1.02 with 95% CI [0.19, 5.38]), and TNK 0.4 (RR: 1.66 with 95% CI [0.90, 3.07]) (Table 3, Figures S12-D, S19, S20). No significant heterogeneity was observed (I^2^ = 17%).

#### Any parenchymal hematoma

In the pairwise meta-analysis, we found no difference between TNK and alteplase (RR: 1.13 with 95% CI [0.83, 1.54], P = 0.44) (very low-quality evidence) (Fig. 4-E, Table S3). Pooled studies were heterogenous (P = 0.03, I^2^ = 59%). Heterogeneity was best resolved after excluding Kvistad et al. [21] (P = 0.27, I^2^ = 27%); however, after excluding Kvistad et al. [21], there was no difference between TNK and alteplase (RR: 0.95 with 95% CI [0.69, 1.32], P = 0.77) (Table S4).

In network meta-analysis, all other TNK doses showed no statistically significant difference, compared to alteplase, except TNK-0.4, which showed a statistically significant higher risk for hematoma (RR: 7.04 with 95% CI [1.27, 39.08]) (Table 3, Figures S12-E, S21, S22). No significant heterogeneity was observed (I^2^ = 21%).

## Discussion

Our network meta-analysis involving nine RCTs, and 3707 patients is the most comprehensive and recent study to compare the efficacy & safety of various TNK doses with alteplase. Our pairwise meta-analysis showed that TNK was associated with a higher rate of complete recanalization; however, we found no difference between TNK and alteplase regarding early neurological improvement, excellent neurological recovery, good neurological recovery (functional independence), and complete/partial recanalization. Also, safety outcomes, including mortality, ICH, and parenchymal hematoma, were similar between both groups. Moreover, our network meta-analysis showed medium dose (TNK 0.25) to have significantly higher early neurological improvement compared with alteplase. Finally, the high dose (TNK 0.4) showed a significantly higher risk of developing parenchymal hematomas.

Evidence from the previous meta-analysis is in consensus about the non-inferiority of (TNK 0.25) compared with alteplase supporting our findings [39]. The early neurological improvement can be attributed to the pharmacokinetic properties of TNK, having a long half-life, higher fibrin specificity, and more potent clot dissolution, leading to faster vessel recanalization [40]. Moreover, although we found no significant differences between low-tier TNK doses vs. alteplase in other efficacy and safety outcomes, previous studies have extensively shown that (TNK 0.25) is associated with better imaging-based outcomes, partial/complete recanalization, and higher levels of neurological function, with no increased risk of intracerebral bleeding or mortality, compared with alteplase [20, 41–44]. This has important clinical implications because it paves the road for (TNK 0.25) to safely replace alteplase as the standard of care. Moreover, the rapid, single bolus infusion of TNK allows for a give-and-go strategy, whereby giving dosage requires as short as one minute. Therefore, decreasing the door-in-to-door out time. This is important in remote settings with poor resources that lack access to thrombectomy canters and require ambulances for transporting patients to specialized stroke centers. This contrasts with the drip and ship paradigm for multiple boluses and prolonged infusion of alteplase for up to one hour [39].

However, this non-inferiority of TNK over alteplase is still a matter of debate as our analysis revealed that the high dose (TNK 0.4) is significantly associated with developing parenchymal hematomas. Earlier evidence involving this dosage has remained inconsistent and inconclusive due to the small sample sizes and the few investigating RCTs. Huang et al. [41], in their meta-analysis, identified a potential correlation between drug dose and increased risk of ICH hemorrhage; however, they failed to establish plausibility in the results due to the small sample size (19 patients). The adverse effects of TNK 0.4 are speculated to be caused by the relatively longer serum half-life of the drug compared to alteplase delaying the achievement of homeostasis. For alteplase, multiple infusions can be stopped once signs of ICH are detected, yet no evidence shows significant alterations in clinical outcomes [39].

In contrast, recent data from the NOR-TEST [37], with 549 patients enrolled in the high-dose group (TNK 0.4), showed no increased risk of ICH, or mortality after three months [37]. This is inconsistent with Yogendrakumar et al.’s [45] pooled analysis of EXTEND-IA TNK trials showing higher rates of symptomatic ICH with TNK 0.4 and symptomatic ICH and mortality TNK 0.25 [45]. Furthermore, in contrast to NOR TEST [37], NOR TEST 2-A trial [21] failed to demonstrate the non-inferiority of TNK 0.4 to alteplase in moderate or severe ischaemic stroke [21]. In the modified intention-to-treat population, the favorable functional outcome at three months occurred less frequently in patients allocated TNK 0.4 compared with alteplase [21]. Also, the rates of ICH, poor functional outcome, and mortality were higher in the TNK 0.4 group [21]. Kvistad et al. [21] attributed this difference to age imbalance between the two groups, with an average five years higher in the TNK group, patients in the TNK group were more likely to have a disability (mRS score ≥ 1), and more patients in the TNK group were diagnosed with AIS; however, alteplase group had more stroke mimics with a relatively better prognosis [21, 46]. This is supported by the findings of our sensitivity analysis which significantly favored TNK over alteplase regarding early neurological recovery after excluding Kvistad et al. [21]. Therefore, NOR TEST 2-A [21] constitutes an important determinant of our study findings, which is an inherited limitation from the trial itself. Also, the trial was terminated prematurely due to increased harm to patients [21].

Regarding recanalization, despite our pairwise analysis showing significant success with TNK, our network meta-analysis showed no difference. This can be attributed to that different treatment groups are underpowered to show statistical differences. Moreover, Parsons et al. [38] supported the theory that there might be an improved recanalization with increasing TNK doses. Still, there is no evidence regarding recanalization with TNK 0.4; hence, more research is required to prove these dose-related claims, as the recanalization rate is a key indicator for improved outcomes [39].

Intravenous thrombolysis by IV alteplase (0.9 mg/kg, maximum dose 90 mg over 60 min with initial 10% of dose given as bolus over 1 min), on one hand, is the only endorsed systemic reperfusion treatment for patients with AIS according to the 2019 American Heart Association/American Stroke Association (AHA/ASA) Guidelines for emergency management for AIS [7]. AHA/ASA recommended alteplase for selected patients who can be treated within a time window of (< 4.5 h) [7]. Similarly, alteplase administered within 4.5 h of symptom recognition can be beneficial in patients with wake-up stroke, having unclear time onset of stroke (> 4.5 h), or diffusion-weighted magnetic resonance imaging (DW-MRI) lesion smaller than $1/3$ of middle cerebral artery territory with no visible change in fluid-attenuated inversion recovery (FLAIR) [7].

On the other hand, AHA/ASA and European Stroke Organization (ESO) stated that TNK (single IV bolus 0.25 mg/kg maximum 25 mg) may be favored over alteplase in patients without contraindications for IV fibrinolysis and are eligible to undergo mechanical thrombectomy as a bridging therapy [7, 47]. However, the quality of evidence for TNK recommendations remains low, and they recommended further RCTs for a conclusive statement regarding TNK [47]. Accordingly, the addition of the recent findings, especially from the AcT trial [22], can strengthen the evidence about TNK 0.25 to replace alteplase for AIS presenting within 4.5 h [48]. Nonetheless, evidence about TNK’s role in disabling stroke presenting after 4.5 h, wake-up stroke, and minor stroke/TIA is still inconclusive.

Regarding disabling stroke presenting after 4.5 h, ESO guidelines recommend alteplase in patients with AIS presenting after up to nine hours after symptoms start with target mismatch (penumbra: potentially rescuable hypoperfused tissue) on CT perfusion imaging and in whom MT is not planned [47, 48]. In this regard, the TIMELESS trial [49], the ROSE-TNK trial [50], and the ETERNAL trial [51] are currently undergoing to compare TNK 0.25 versus alteplase in AIS presenting beyond 4.5 h along with target mismatch [48].

Similarly, ESO guidelines recommend alteplase for wake-up stroke, provided that the patient fulfills certain imaging criteria [47]. In this regard, the TWIST trial [47] tested TNK 0.25 versus no thrombolysis for patients with wake-up stroke presenting within 4.5 h from awakening; however, it was prematurely terminated and thus underpowered to test the non-inferiority or superiority of TNK over alteplase [48].

Despite that minor stroke’s definition is still controversial with no clear distinguishing between disabling and non-disabling symptoms by currently used scores, such as NIHSS [48], AHA/ASA, and ESO guidelines recommended alteplase for minor stroke with disabling symptoms [7, 47]. Furthermore. AHA/ASA recommended TNK 0.4 for minor stroke based on the NORTEST-1 trial [37], which may not continue after NORTEST-2A [21]. In this regard, the TEMPO-1 trial, a dose escalation trial of TNK in minor stroke/TIA (NIHSS 0–5), found that TNK 0.1 and 0.25 are safe [52]. Currently, the TEMPO-2 trial is comparing TNK 0.25 versus standard of care in patients with minor stroke or TIA who have a confirmed LVO [53].

Notably, all of the included trials were prospective, randomized, open-label, and blinded outcome (PROBE) trials except Haley et al. 2010 [36], which was double-blinded RCT; however, prematurely terminated. Moreover, all the ongoing trials are PROBE trials except the TIMELESS trial, which may provide more subtle results [49, 54]. PROBE trials fail to overcome information bias which may lead to unconvincing outcomes as observed by our GRADE assessment [54]. Hence, future studies should consider the double-blinded design. Furthermore, RCTs that have the potential to reshape management and enhance outcomes for stroke patients are resource-demanding [55], and stroke research funding is considerably lower compared to cancer and heart research [55, 56]. Therefore, the pragmatic design of future RCTs following the AcT trial [22] can decrease the required funding and time needed to register the same factors into variable databases [55]. Finally, using wide inclusion criteria (any AIS patient eligible for thrombolysis), deferred consents, and a simple randomization process are also required in future RCTs, given the time-restricted nature of AIS management [55].

## Strengths & Limitations

Our study is the most comprehensive and up-to-date network meta-analysis synthesizing only RCTs constituting the gold standard evidence in this regard. However, our review has a few limitations: first, most of the included RCTs are open-label trials with a high risk of performance bias. Second, the results should be interpreted with caution since the included trials differed in aspects such as advanced imaging for patient selection, presence of large vessel occlusion, the time window for drug administration or endovascular therapy, and variation in patient populations making indirect comparisons less conclusive. Finally, some of our findings show significant heterogeneity, which can limit the generalizability of our results.

## Implications for Future Research

Considering the above discussion, rigorous double-blinded, pragmatic RCTs are required to investigate TNK’s potential to replace alteplase as the standard of care in AIS presenting after 4.5 h from symptoms onset, wake-up stroke, and minor stroke/TIA. Furthermore, future RCTs should consider investigating: recanalization time with dose escalation, extending safety outcome measurements to include systematic bleeding events, cerebral infarction in a new vascular area, and vessel re-occlusion [54], other predictors of the stroke care pathway, such as door to groin time, and efficacy of TNK as a bridging therapy before MT. Moreover, all of the completed and ongoing trials are from high-income regions with Caucasian ethnicity predominance; thus, RCTs in low- and middle-income regions and different ethnicities are still required [54]. Finally, cost benefits and drug administration techniques of TNK versus alteplase can also be assessed as considerations in this area that would be valuable for universal access to stroke care in low and middle-income countries.

## Conclusion

TNK in the dose of 0.25 mg is a promising candidate to replace alteplase as the standard of care in patients with AIS presenting within 4.5 h of symptom onset, given its higher rate of early neurological recovery and non-inferiority in terms of safety outcomes. However, the evidence regarding TNK’s potential in AIS presenting after 4.5 h from symptoms onset, wake-up stroke, and minor stroke/TIA is still lacking, which accordingly warrants conducting further double-blinded, large-scale, and pragmatic RCTs.

## Supplementary Information

Below is the link to the electronic supplementary material.

Supplementary file1 (DOCX 4106 KB)

## Data Availability

Not applicable.
