# Supplementary material for: The efficacy and safety of tenecteplase versus alteplase for acute ischemic stroke: an updated systematic review, pairwise, and network meta-analysis of randomized controlled trials
Source: J Thromb Thrombolysis. 2022 Nov 30;55(2):322–38. doi: 10.1007/s11239-022-02730-5 (PMC10011306; doi:10.1007/s11239-022-02730-5)
Supplement: Supplementary file 1 — Supplementary file1 (DOCX 4106 KB) [file 11239_2022_2730_MOESM1_ESM.docx]

**Title.**

**The Efficacy and Safety of Tenecteplase versus Alteplase for Acute Ischemic Stroke: An Updated Systematic Review, Pairwise, and Network Meta-Analysis of Randomized Controlled Trials.**

**Running Title.**Tenecteplase versus Alteplase for Stroke.

**Authors.**

Mohamed Abuelazm^1^, Amith Reddy Seri^2,3^, Ahmed K. Awad^4^, Unaiza Ahmed^5^, Abdelrahman Mahmoud^6^, Ebraheem Albazee^7^, Soumya Kambalapalli^2,3^, Basel Abdelazeem^2,3^.

**Affiliations.**

1. Faculty of Medicine, Tanta University, Tanta, Egypt.
2. Department of Internal Medicine, McLaren Health Care, Flint, Michigan, USA.
3. Department of Internal Medicine, Michigan State University, East Lansing, Michigan, USA.
4. Faculty of Medicine, Ain-Shams University, Cairo, Egypt.
5. Punjab Medical College, Faisalabad, Pakistan.
6. Faculty of Medicine, Minia University, Minia, Egypt.
7. Kuwaite Institute for Medical Specializations (KIMS), Kuwait City, Kuwait.

**Keywords.**

Tenecteplase; Alteplase; Stroke; Systematic Review; Meta-Analysis; Controlled Trials.

**Corresponding author.**

Amith Reddy Seri, MBBS

Department of Internal Medicine

McLaren Health Care/Michigan State University

Flint, Michigan, USA.

Email : [seriamithreddy@gmail.com](mailto:seriamithreddy@gmail.com)

Tel: +15202727116

**Contents:**

**Tables.**

Table S1: Search terms and results in different databases.

Table S2: Author judgments for ROB assessment.

Table S3: GRADE evidence profile.

Table S4: Sensitivity analysis.

**Figures.**

Figure S1: Forest plot of pooled summary estimates derived from network meta-analysis (A- early neurological improvement, B- excellent neurological recovery, C- good neurological recovery, D- complete recanalization, and E- partial/complete recanalization

Figure S2: Network plot of early neurological improvement.

Figure S3: Forest plot of individual study results grouped by treatment component for early neurological improvement.

Figure S4: Network plot of excellent neurological recovery.

Figure S5: Forest plot of individual study results grouped by treatment component for excellent neurological improvement.

Figure S6: Network plot of good neurological recovery.

Figure S7: Forest plot of individual study results grouped by treatment component for good neurological improvement.

Figure S8: Network plot of complete recanalization.

Figure S9: Forest plot of individual study results grouped by treatment component for complete recanalization.

Figure S10: Network plot of partial/complete recanalization.

Figure S11: Forest plot of individual study results grouped by treatment component for partial/complete recanalization.

Figure S11: Network plot of poor neurological recovery.

Figure S12 Forest plot of pooled summary estimates derived from network meta-analysis (A- poor neurological improvement, B- all-cause mortality at 90 days, C- any intracranial hemorrhage, D- symptomatic intracranial hemorrhage, and E- any parenchymal hematoma), TNK: tenecteplase, RR: risk ratio, CI: confidence interval.

Figure S13: Forest plot of individual study results grouped by treatment component for poor neurological recovery.

Figure S14: Network plot of all-cause mortality.

Figure S15 Forest plot of individual study results grouped by treatment component for all-cause mortality.

Figure S16: Network plot of any intracranial hemorrhage.

Figure S17: Forest plot of individual study results grouped by treatment component for any intracranial hemorrahge.

Figure S18: Network plot of symptomatic intracranial hemorrhage.

Figure S19: Forest plot of individual study results grouped by treatment component for symptomatic intracranial hemorrahge.

Figure S20: Network plot of any parenchymal hematoma.

Figure S21: Forest plot of individual study results grouped by treatment component for any parenchymal hematoma.

| Database | Search Terms | Search Field | Search Results |
| --- | --- | --- | --- |
| PubMed | (Tenecteplase OR TNK-tPA OR TNK OR metalyse OR TNKase OR "recombinant human TNK tissue-type plasminogen activator*" OR  rhTNK-tPA) AND (alteplase OR "tissue plasminogen activator*" OR "(Tissue Type Plasminogen Activator*" OR "T-Plasminogen Activator" OR rt-PA) AND (stroke OR "cerebrovascular Accident*" OR "Brain Vascular Accident*" OR "acute stroke*") | All Fields | 278 |
| Cochrane | (Tenecteplase OR TNK-tPA OR TNK OR metalyse OR TNKase OR "recombinant human TNK tissue-type plasminogen activator*" OR  rhTNK-tPA) AND (alteplase OR "tissue plasminogen activator*" OR "(Tissue Type Plasminogen Activator*" OR "T-Plasminogen Activator" OR rt-PA) AND (stroke OR "cerebrovascular Accident*" OR "Brain Vascular Accident*" OR "acute stroke*") | All Fields | 194 |
| WOS | (Tenecteplase OR TNK-tPA OR TNK OR metalyse OR TNKase OR "recombinant human TNK tissue-type plasminogen activator*" OR  rhTNK-tPA) AND (alteplase OR "tissue plasminogen activator*" OR "(Tissue Type Plasminogen Activator*" OR "T-Plasminogen Activator" OR rt-PA) AND (stroke OR "cerebrovascular Accident*" OR "Brain Vascular Accident*" OR "acute stroke*") | All Fields | 328 |
| EMBASE | #4.  #1 AND #2 AND #3                                           374  #3.  stroke:ti,ab,kw OR 'cerebrovascular accident':ti,ab,kw OR 'brain vascular accident':ti,ab,kw OR 'acute stroke':ti,ab,kw                   474,189  #2.  alteplase:ti,ab,kw OR 'tissue plasminogen activator*':ti,ab,kw OR 'tissue type plasminogen  activator*':ti,ab,kw OR 't-plasminogen activator':ti,ab,kw OR 'rt pa':ti,ab,kw             31,559  #1.  tenecteplase:ti,ab,kw OR 'tnk tpa':ti,ab,kw OR tnk:ti,ab,kw OR metalyse:ti,ab,kw OR  tnkase:ti,ab,kw OR 'recombinant human tnk tissue-type plasminogen activator*':ti,ab,kw OR 'rhtnk tpa':ti,ab,kw   1,436 | All Fields | 374 |
| SCOPUS | TITLE-ABS-KEY ( ( tenecteplase  OR  tnk-tpa  OR  tnk  OR  metalyse  OR  tnkase  OR  "recombinant human TNK tissue-type plasminogen activator*"  OR  rhtnk-tpa )  AND  ( alteplase  OR  "tissue plasminogen activator*"  OR  "(Tissue Type Plasminogen Activator*"  OR  "T-Plasminogen Activator"  OR  rt-pa )  AND  ( stroke  OR  "cerebrovascular Accident*"  OR  "Brain Vascular Accident*"  OR  "acute stroke*" ) ) | Title, Abstract, Keywords | 710 |

Table S1: Search terms and results in different databases.

| Study ID | Domain | Judgment |
| --- | --- | --- |
| Bivard et al. 2022 | Performance bias | High-risk “open-label trial” |
| Campbell et al. 2018 | Performance bias | High-risk “open-label trial” |
| Haley et al. 2010 | Selection bias | Unclear risk “data on allocation not available” |
| Huang et al. 2015 | Performance bias | High-risk “open-label trial” |
| Kvistad et al. 2022 | Performance bias | High-risk “open-label trial” |
| Li et al. 2021 | Selection bias | High-risk “open-label trial with no concealment” |
|  | Performance bias | High-risk “open-label trial” |
|  | Other bias | High risk “This study was sponsored and funded by Guangzhou Recomgen Biotech Co., Ltd.” |
| Logallo et al. 2017 | Performance bias | High-risk “open-label trial” |
| Menon et al. 2022 | Performance bias | High-risk “open-label trial” |
| Parsons et al. 2012 | Performance bias | High-risk “open-label trial” |
|  | Other bias | Unclear risk “although the study was Funded by the Australian National Health and Medical Research Council, there was no pharmaceutical companies funding the study”. |

Table S2: Author judgments for ROB assessment.

| **Certainty assessment** | | | | | | | **№ of patients** | | **Effect** | | **Certainty** |
| --- | --- | --- | --- | --- | --- | --- | --- | --- | --- | --- | --- |
| **№ of studies** | **Study design** | **Risk of bias** | **Inconsistency** | **Indirectness** | **Imprecision** | **Other considerations** | **[intervention]** | **[comparison]** | **Relative (95% CI)** | **Absolute (95% CI)** |  |
| **Early neurological improvement** | | | | | | | | | | | |
| 7 | randomised trials | serious^a^ | serious^b^ | not serious | not serious | none | 715/1745 (41.0%) | 545/1530 (35.6%) | **RR 1.09** (1.01 to 1.19) | **32 more per 1,000** (from 4 more to 68 more) | ⨁⨁◯◯ Low |
| **Excellent neurological recovery (mRS 0–1)** | | | | | | | | | | | |
| 9 | randomised trials | very serious^a^ | not serious | not serious | not serious | none | 930/1958 (47.5%) | 791/1731 (45.7%) | **RR 1.03** (0.96 to 1.10) | **14 more per 1,000** (from 18 fewer to 46 more) | ⨁⨁◯◯ Low |
| **Good neurological recovery (mRS 0–2)** | | | | | | | | | | | |
| 8 | randomised trials | very serious^a^ | serious^b^ | not serious | not serious | none | 1193/1877 (63.6%) | 1080/1700 (63.5%) | **RR 1.00** (0.89 to 1.13) | **0 fewer per 1,000** (from 70 fewer to 83 more) | ⨁◯◯◯ Very low |
| **Compelete/Partial Recanalization** | | | | | | | | | | | |
| 4 | randomised trials | serious^a^ | very serious^b^ | not serious | serious^c^ | none | 129/214 (60.3%) | 98/194 (50.5%) | **RR 1.12** (0.82 to 1.54) | **61 more per 1,000** (from 91 fewer to 273 more) | ⨁◯◯◯ Very low |
| **Compelete Recanalization** | | | | | | | | | | | |
| 4 | randomised trials | serious^a^ | not serious | not serious | serious^c^ | none | 124/435 (28.5%) | 87/405 (21.5%) | **RR 1.27** (1.02 to 1.57) | **58 more per 1,000** (from 4 more to 122 more) | ⨁⨁◯◯ Low |
| **Any-Cause Mortality at 90 days** | | | | | | | | | | | |
| 9 | randomised trials | not serious | not serious | not serious | not serious | none | 217/1952 (11.1%) | 194/1724 (11.3%) | **RR 0.99** (0.82 to 1.18) | **1 fewer per 1,000** (from 20 fewer to 20 more) | ⨁⨁⨁⨁ High |
| **Poor neurological recovery (mRS 4–6)** | | | | | | | | | | | |
| 9 | randomised trials | very serious^a^ | not serious | not serious | not serious | none | 440/1958 (22.5%) | 397/1731 (22.9%) | **RR 0.97** (0.86 to 1.10) | **7 fewer per 1,000** (from 32 fewer to 23 more) | ⨁⨁◯◯ Low |
| **Any intracerebral hemorrhage** | | | | | | | | | | | |
| 7 | randomised trials | serious^a^ | not serious | not serious | not serious | none | 265/1855 (14.3%) | 241/1658 (14.5%) | **RR 1.00** (0.85 to 1.18) | **0 fewer per 1,000** (from 22 fewer to 26 more) | ⨁⨁⨁◯ Moderate |
| **Symptomatic intracerebral hemorrhage** | | | | | | | | | | | |
| 9 | randomised trials | serious^a^ | not serious | not serious | serious^c^ | none | 64/1960 (3.3%) | 48/1732 (2.8%) | **RR 1.15** (0.80 to 1.67) | **4 more per 1,000** (from 6 fewer to 19 more) | ⨁⨁◯◯ Low |
| **Any parenchymal hematoma** | | | | | | | | | | | |
| 6 | randomised trials | serious^a^ | serious^b^ | not serious | serious^c^ | none | 81/1153 (7.0%) | 68/1091 (6.2%) | **RR 1.11** (0.82 to 1.52) | **7 more per 1,000** (from 11 fewer to 32 more) | ⨁◯◯◯ Very low |

Table S3: GRADE evidence profile.

CI: confidence interval; RR: risk ratio

**Explanations:**

a. Included trials show a high risk of performance bias, being open-label trials.

b. I-square > 50%

c. The confidence interval does not exclude the risk of appreciable benefit/harm.

| Outcome | No. of  participants (Tenecteplase/Alteplase) | No. of  trials | Quantitative data synthesis | | | | Heterogeneity analysis | | |
| --- | --- | --- | --- | --- | --- | --- | --- | --- | --- |
|  |  |  | RR | 95% CI | Z value | p-value | df | p-value | I2 (%) |
|  | | | | | | | | | |
| **Early Neurological Improvement** | | | | | | | | | |
| All Studies | 1836/1628 | 8 | 1.07 | [0.94, 1.21] | 0.98 | 0.33 | 7 | 0.04 | 53 |
| Omitting  Campbell et al. 2018 | 1735/1527 | 7 | 1.08 | [0.92, 1.27] | 0.99 | 0.32 | 6 | 0.02 | 60 |
| Omitting  Haley et al. 2010 | 1755/1597 | 7 | 1.06 | [0.93, 1.20] | 0.83 | 0.41 | 6 | 0.03 | 56 |
| Omitting  Huang et al. 2015 | 1789/1579 | 7 | 1.05 | [0.92, 1.18] | 0.7 | 0.49 | 6 | 0.05 | 52 |
| Omitting  Kvistad et al. 2022 | 1748/1530 | 7 | 1.09 | [1.01, 1.19] | 2.08 | 0.04 | 6 | 0.41 | 1 |
| Omitting  Li et al. 2021 | 1659/1569 | 7 | 1.07 | [0.92, 1.25] | 0.86 | 0.39 | 6 | 0.02 | 59 |
| Omitting  Logallo et al. 2017 | 1287/1077 | 7 | 1.08 | [0.91, 1.28] | 0.92 | 0.36 | 6 | 0.02 | 59 |
| Omitting  Menon et al. 2022 | 1096/914 | 7 | 1.08 | [0.92, 1.28] | 0.95 | 0.34 | 6 | 0.02 | 60 |
| Omitting  Parsons et al. 2012 | 1786/1603 | 7 | 1.04 | [0.92, 1.17] | 0.62 | 0.54 | 6 | 0.08 | 47 |
| **Good Neurological Recovery** | | | | | | | | | |
| All Studies | 1877/1700 | 8 | 1.00 | [0.89, 1.13] | 0.06 | 0.95 | 7 | 0.001 | 70 |
| Omitting  Bivard et al. 2022 | 1822/1651 | 7 | 0.98] | [0.87, 1.11 | 0.25 | 0.81 | 6 | 0.001 | 72 |
| Omitting  Campbell et al. 2018 | 1776/1599 | 7 | 0.97 | [0.86, 1.10] | 0.44 | 0.66 | 6 | 0.003 | 69 |
| Omitting  Huang et al. 2015 | 1830/1651 | 7 | 1.01 | [0.89, 1.14] | 0.12 | 0.9 | 6 | 0.0007 | 74 |
| Omitting  Kvistad et al. 2022 | 1791/1599 | 7 | 1.04 | [0.95, 1.13] | 0.87 | 0.39 | 6 | 0.12 | 41 |
| Omitting  Li et al. 2021 | 1700/1641 | 7 | 1.01 | [0.88, 1.16] | 0.2 | 0.84 | 6 | 0.0007 | 74 |
| Omitting  Logallo et al. 2017 | 1328/1149 | 7 | 1.02 | [0.86, 1.22] | 0.23 | 0.82 | 6 | 0.0008 | 74 |
| Omitting  Menon et al. 2022 | 1075/935 | 7 | 1.01 | [0.85, 1.20] | 0.13 | 0.89 | 6 | 0.0008 | 74 |
| Omitting  Parsons et al. 2012 | 1827/1675 | 7 | 0.98 | [0.87, 1.10] | 0.37 | 0.71 | 6 | 0.004 | 69 |
| **Complete/Partial Recanalization** | | | | | | | | | |
| All Studies | 214/194 | 4 | 1.12 | [0.82, 1.54] | 0.71 | 0.48 | 3 | 0.001 | 81 |
| Omitting  Bivard et al. 2022 | 179/159 | 3 | 1.20 | [0.85, 1.69] | 1.02 | 0.31 | 2 | 0.07 | 62 |
| Omitting  Campbell et al. 2018 | 117/95 | 3 | 1.04 | [0.81, 1.34] | 0.30 | 0.76 | 2 | 0.04 | 69 |
| Omitting  Huang et al. 2015 | 182/159 | 3 | 1.24 | [0.70, 2.18] | 0.75 | 0.46 | 2 | 0.0001 | 91 |
| Omitting  Parsons et al. 2012 | 164/169 | 3 | 1.04 | [0.76, 1.43] | 0.24 | 0.81 | 2 | 0.02 | 75 |
| **Any Parenchymal Hematoma** | | | | | | | | | |
| All Studies | 1153/1091 | 6 | 1.11 | [0.82, 1.52] | 0.69 | 0.49 | 5 | 0.04 | 58 |
| Omitting  Bivard et al. 2022 | 1098/1042 | 5 | 1.12 | [0.82, 1.53] | 0.70 | 0.48 | 4 | 0.02 | 66 |
| Omitting  Campbell et al. 2018 | 1052/990 | 5 | 1.11 | [0.80, 1.53] | 0.63 | 0.53 | 4 | 0.02 | 66 |
| Omitting  Huang et al. 2015 | 1105/1042 | 5 | 1.18 | [0.86, 1.62] | 1.04 | 0.3 | 4 | 0.05 | 59 |
| Omitting  Kvistad et al. 2022 | 1053/987 | 5 | 0.95 | [0.69, 1.32] | 0.29 | 0.77 | 4 | 24 | 27 |
| Omitting  Menon et al. 2022 | 353/328 | 5 | 1.19 | [0.67, 2.10] | 0.58 | 0.56 | 4 | 0.02 | 67 |
| Omitting  Parsons et al. 2012 | 1103/1066 | 5 | 1.20 | [0.87, 1.65] | 1.11 | 0.27 | 4 | 0.08 | 52 |

Table S4: Sensitivity analysis.

CI: confidence interval; df: degrees of freedom; RR: risk ratio

***
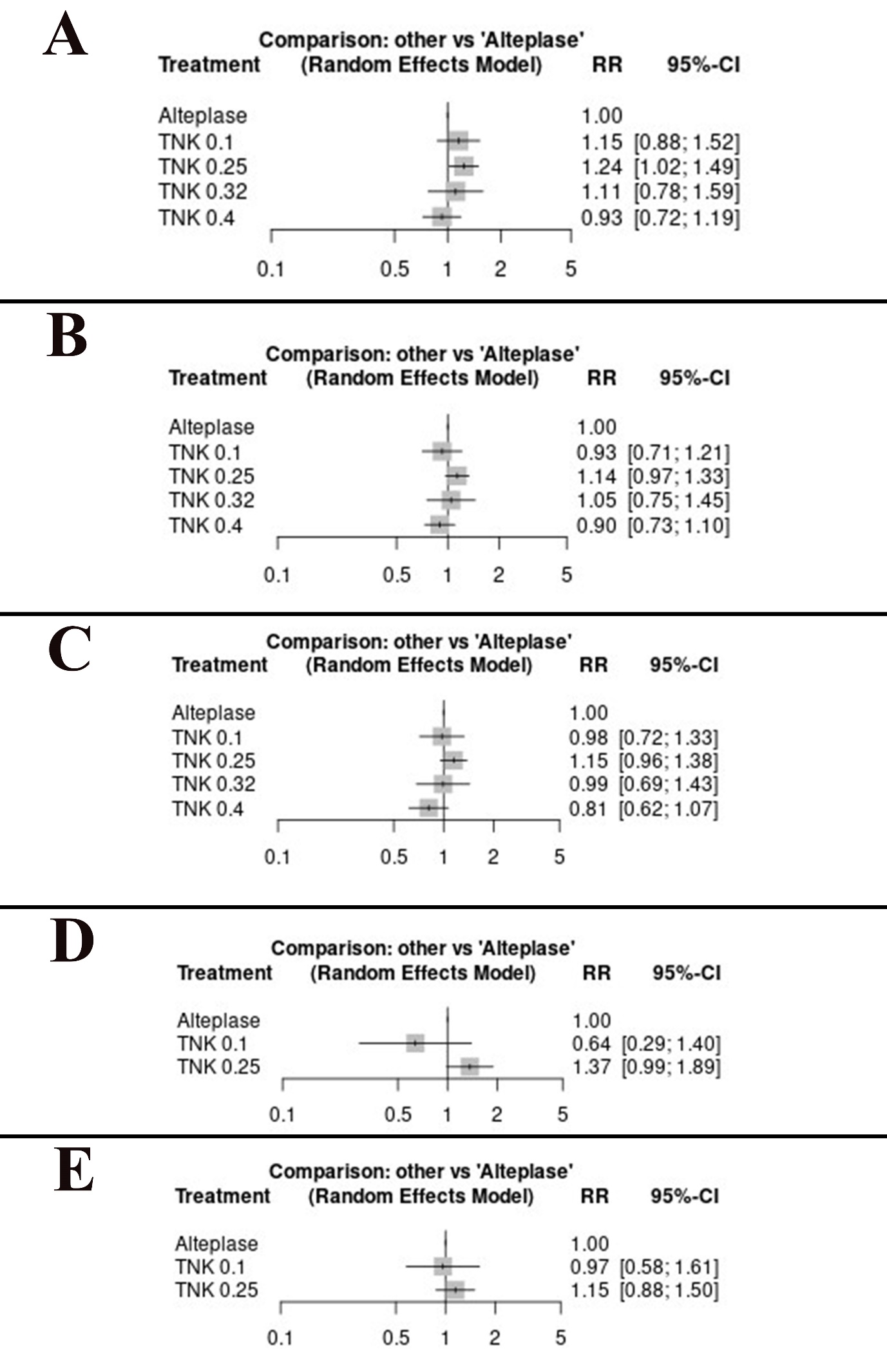
***

Figure S1: Forest plot of pooled summary estimates derived from network meta-analysis (A- early neurological improvement, B- excellent neurological recovery, C- good neurological recovery, D- complete recanalization, and E- partial/complete recanalization

Figure S2: Network plot of early neurological improvement.


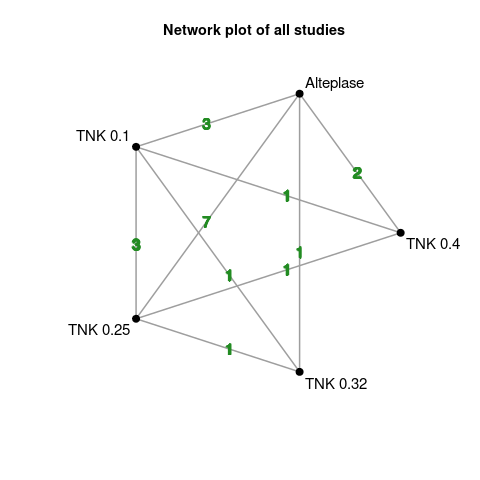


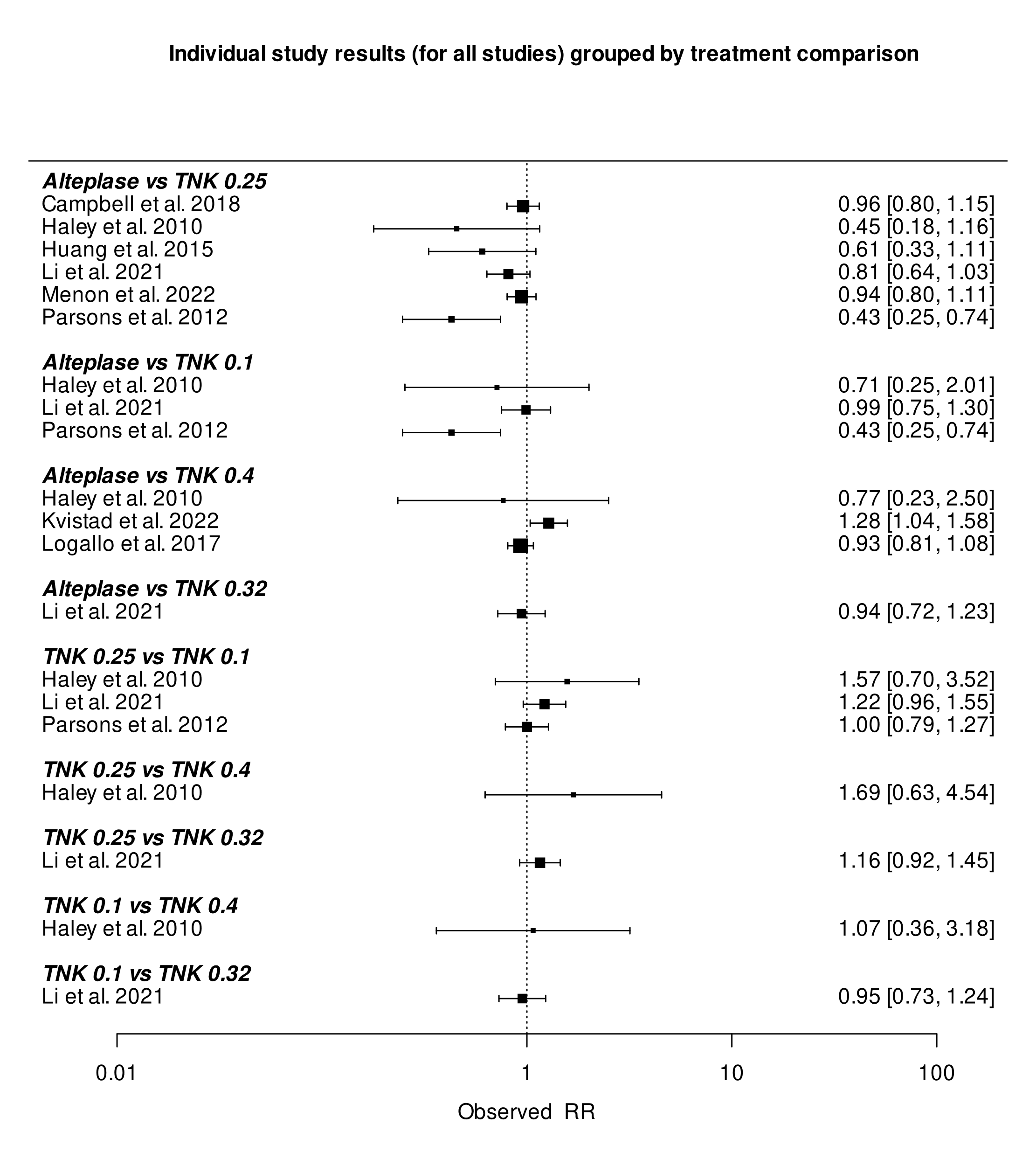


Figure S3: Forest plot of individual study results grouped by treatment component for early neurological improvement.


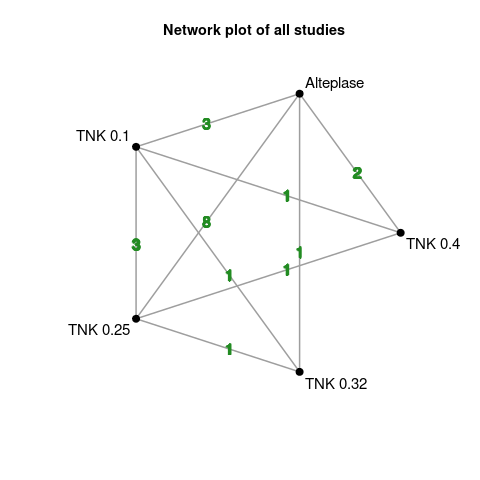

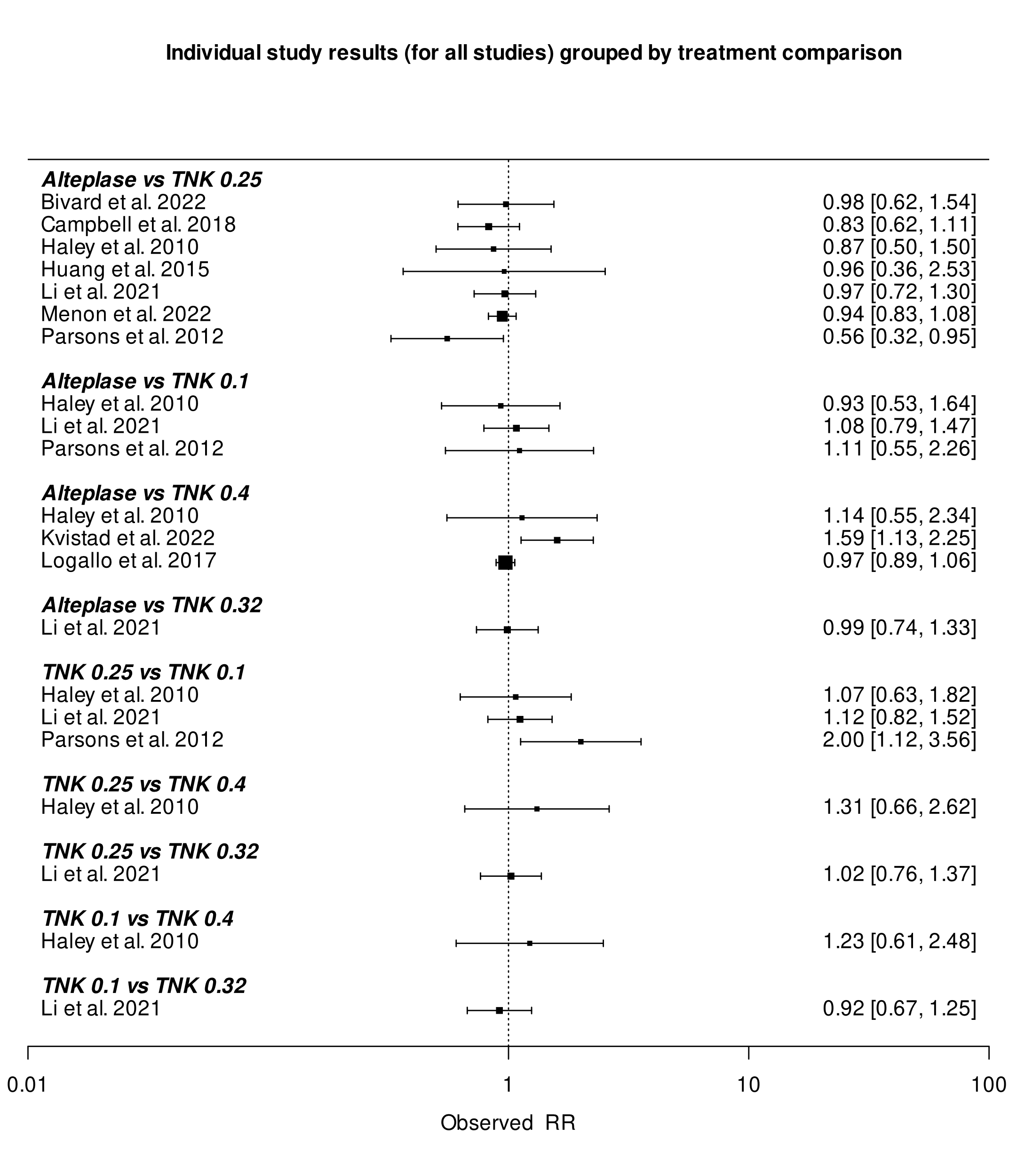


Figure S4: Network plot of excellent neurological recovery.

Figure S5: Forest plot of individual study results grouped by treatment component for excellent neurological improvement.


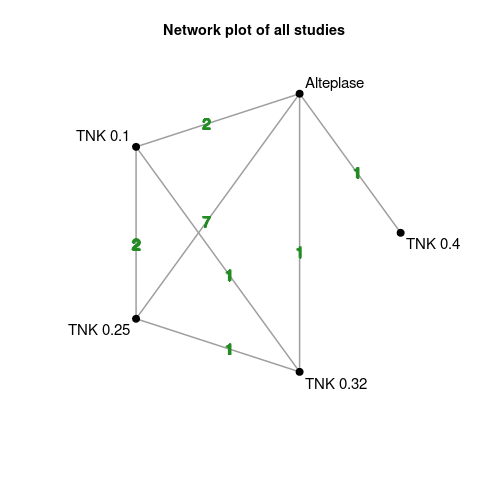


Figure S6: Network plot of good neurological recovery.


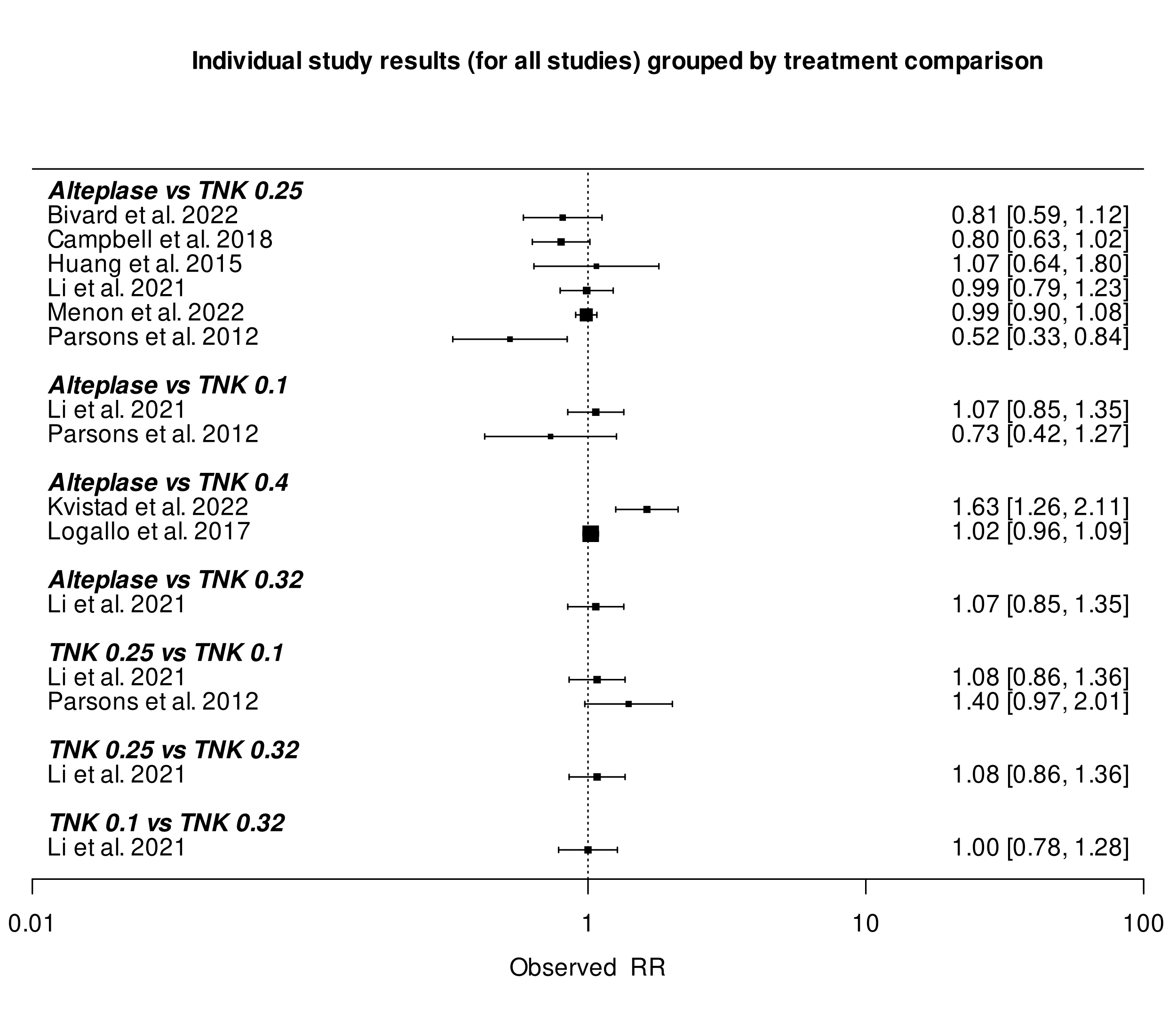


Figure S7: Forest plot of individual study results grouped by treatment component for good neurological improvement.


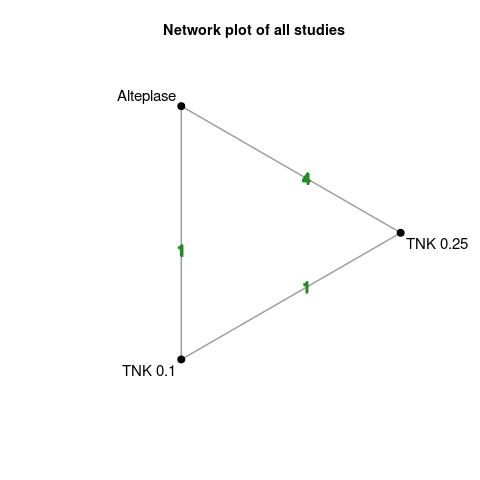


Figure S8: Network plot of complete recanalization.


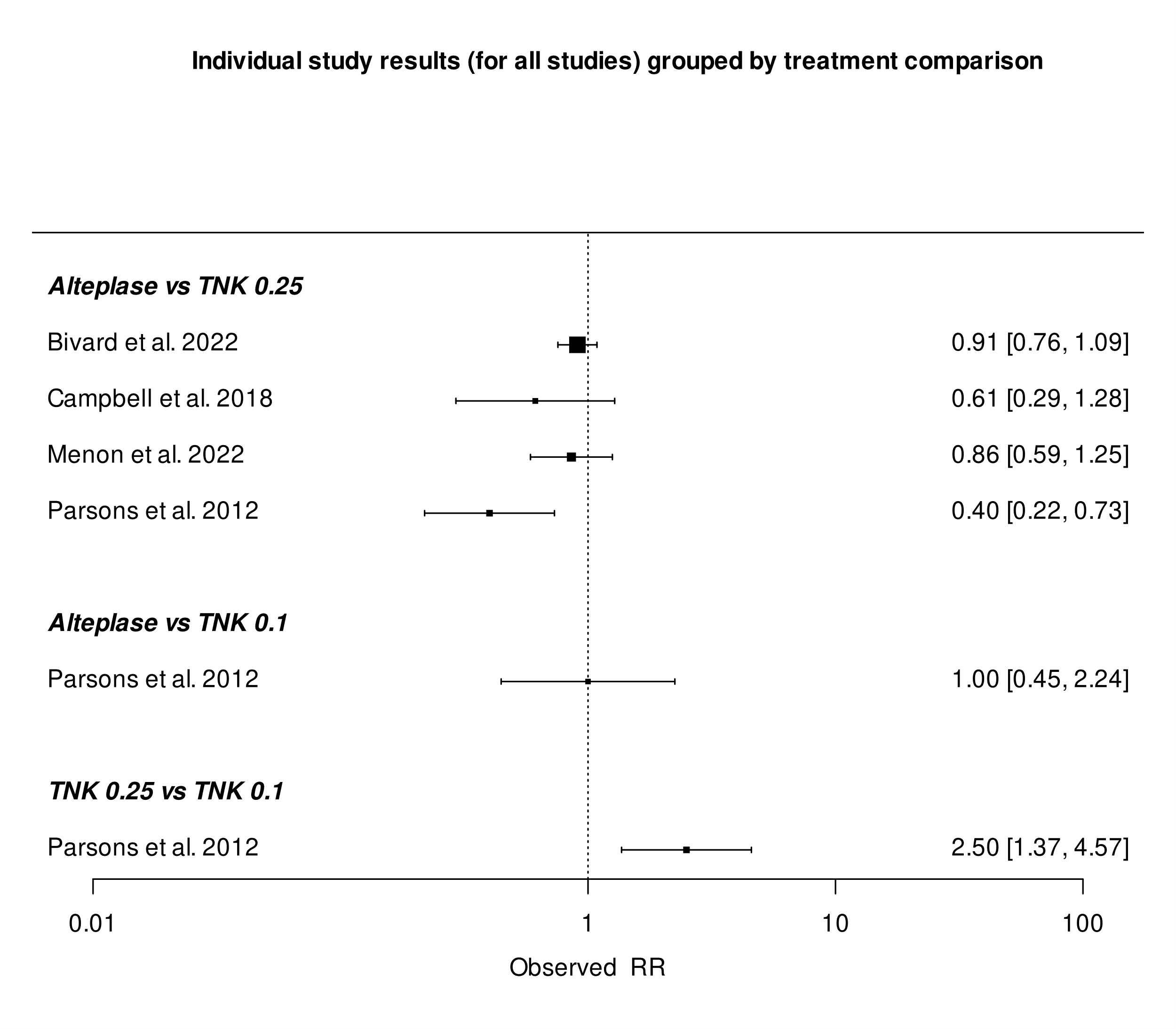


Figure S9: Forest plot of individual study results grouped by treatment component for complete recanalization.


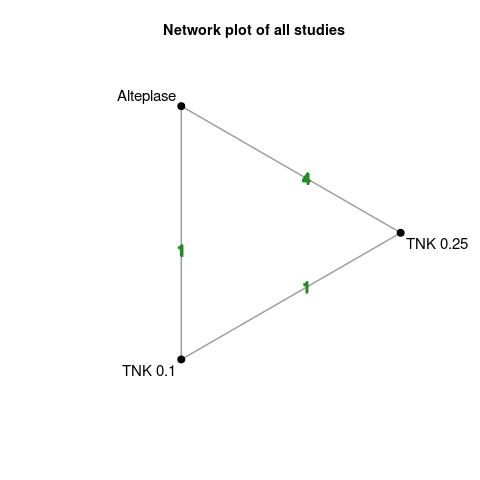


Figure S10: Network plot of partial/complete recanalization.

Figure S11: Forest plot of individual study results grouped by treatment component for partial/complete recanalization.


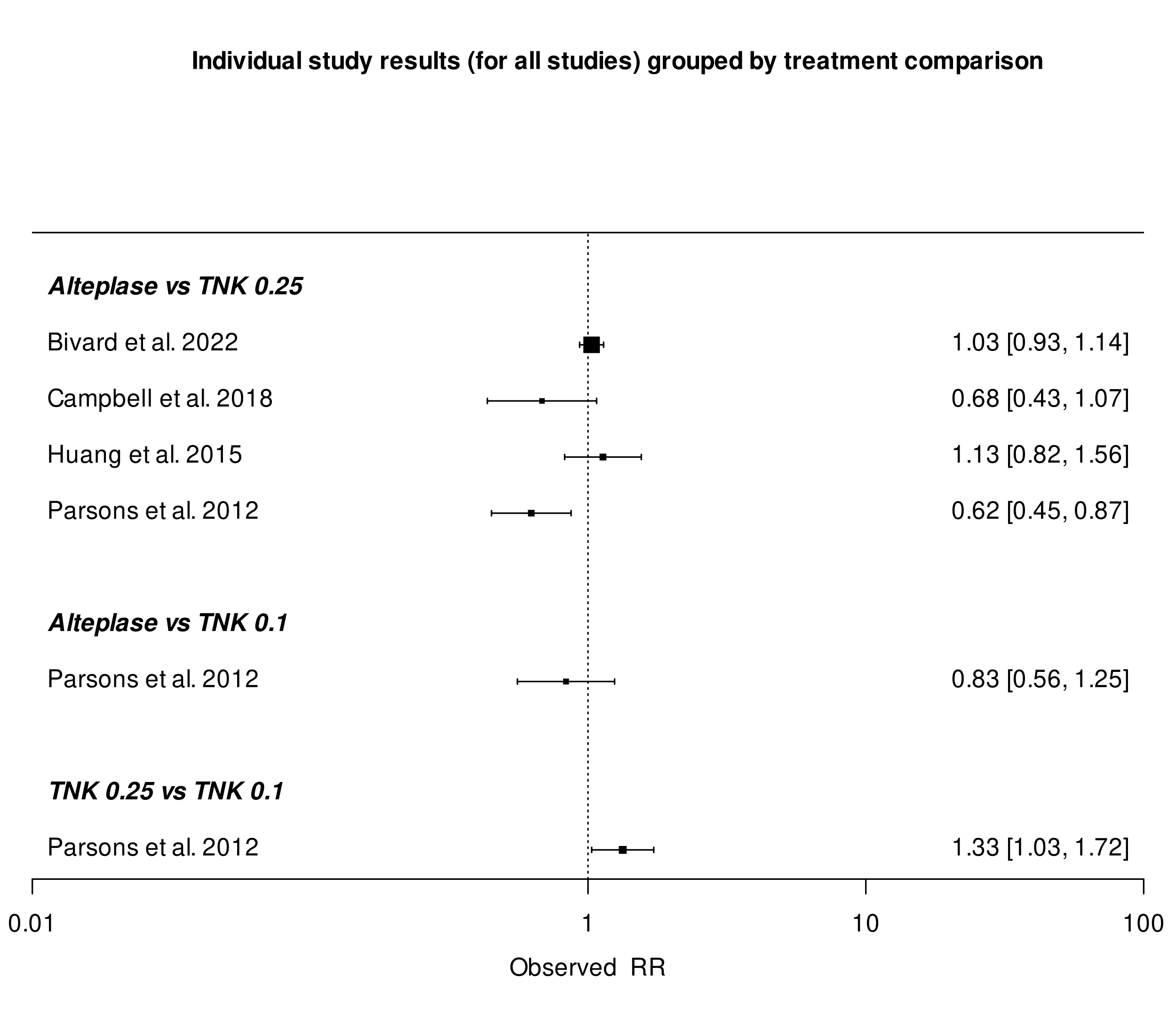


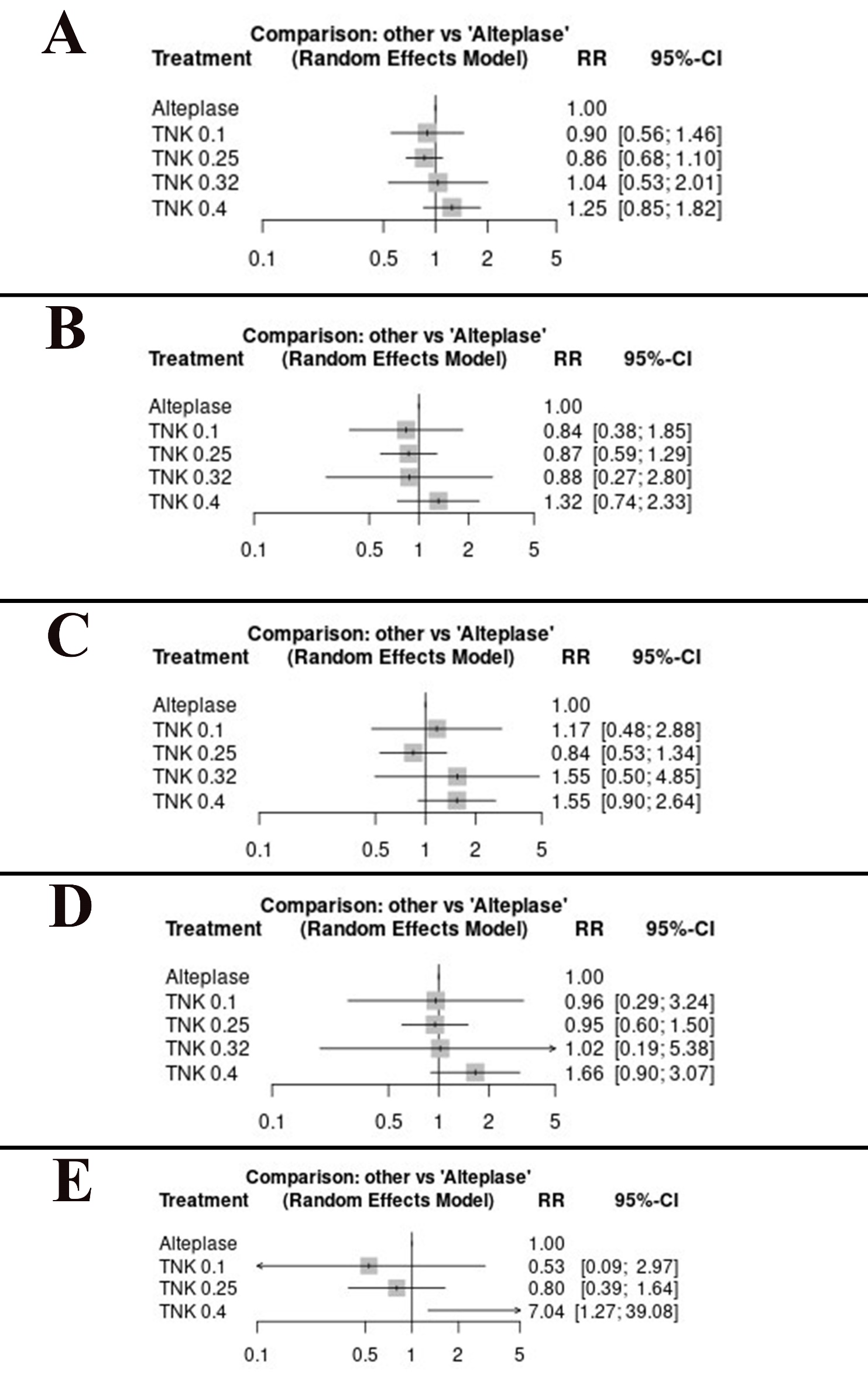

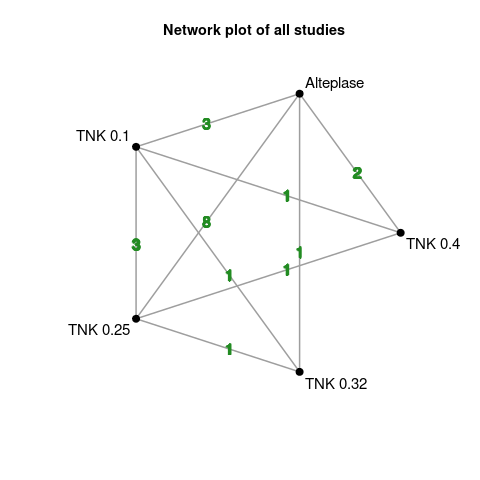


Figure S12 Forest plot of pooled summary estimates derived from network meta-analysis (A- poor neurological improvement, B- all-cause mortality at 90 days, C- any intracranial hemorrhage, D- symptomatic intracranial hemorrhage, and E- any parenchymal hematoma), TNK: tenecteplase, RR: risk ratio, CI: confidence interval.

Figure S13: Network plot of poor neurological recovery.


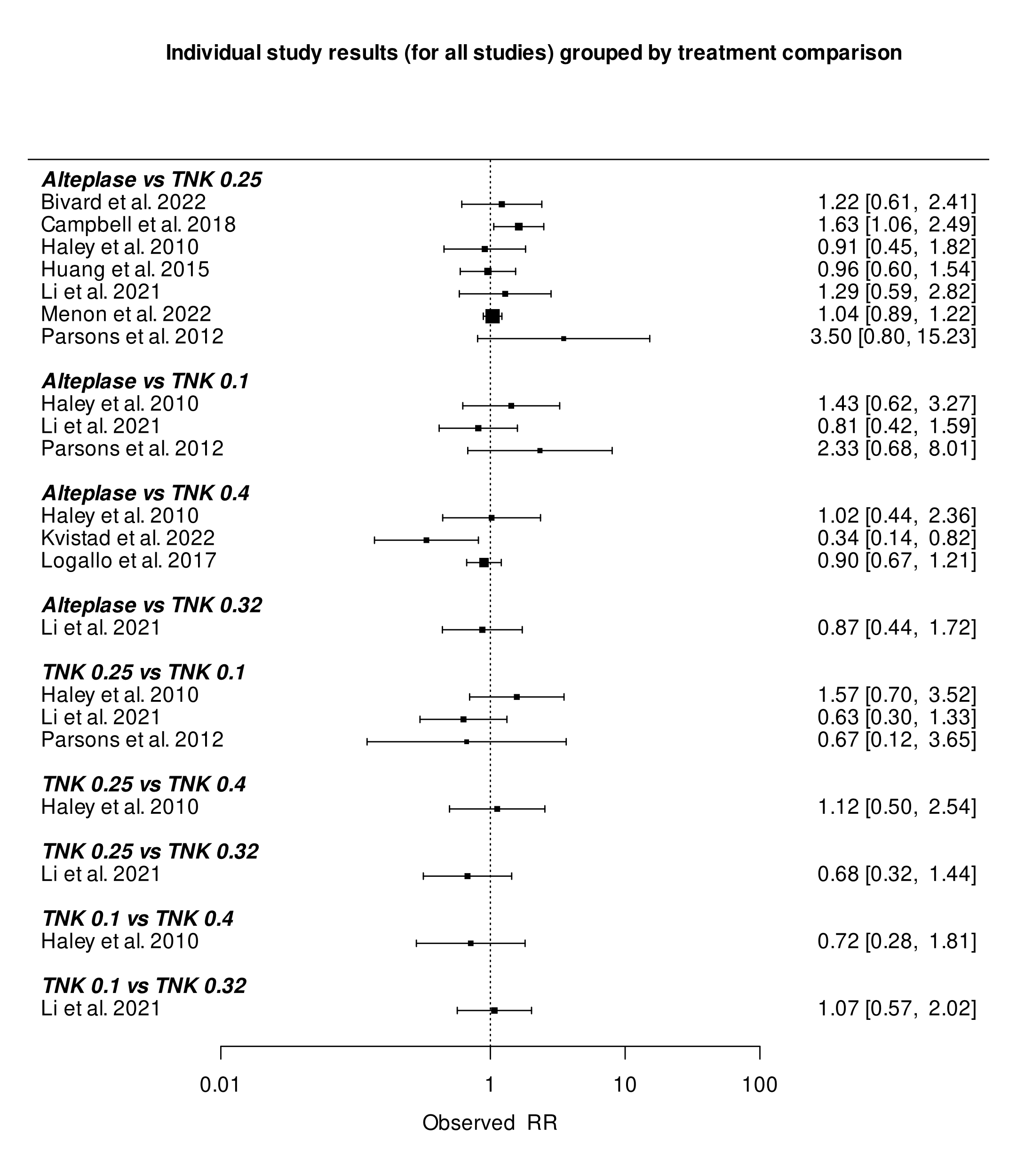


Figure S14: Forest plot of individual study results grouped by treatment component for poor neurological recovery.


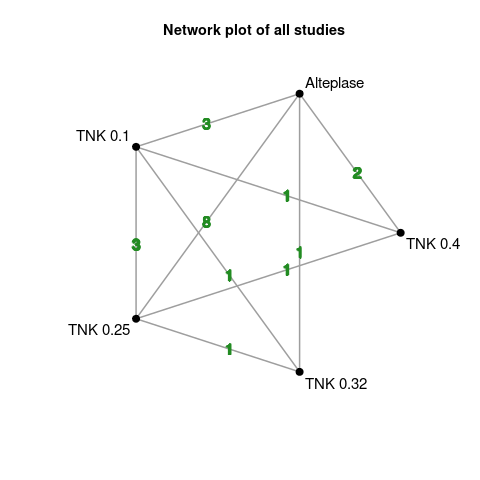


Figure S15: Network plot of all-cause mortality.


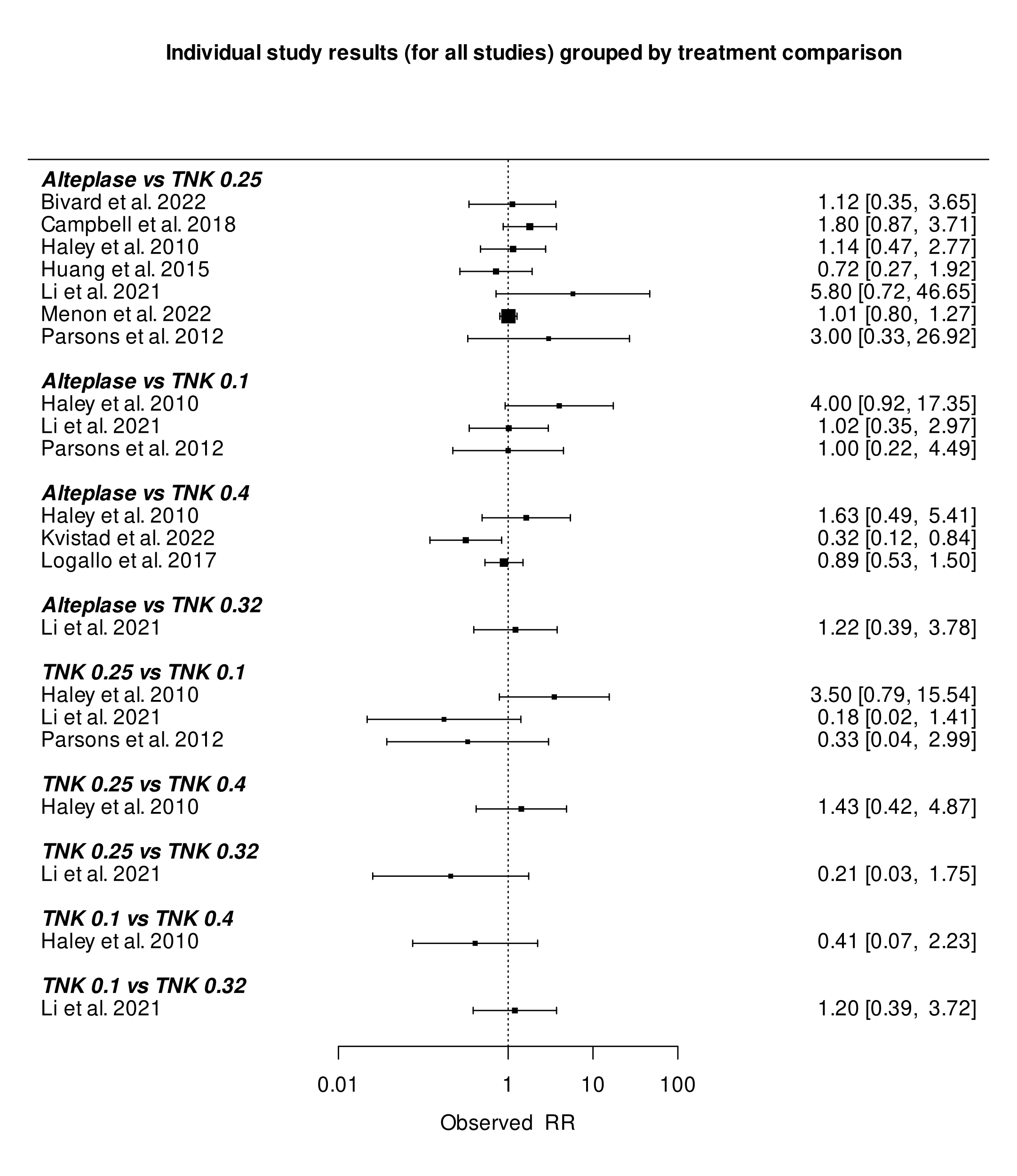


Figure S16: Forest plot of individual study results grouped by treatment component for all-cause mortality.


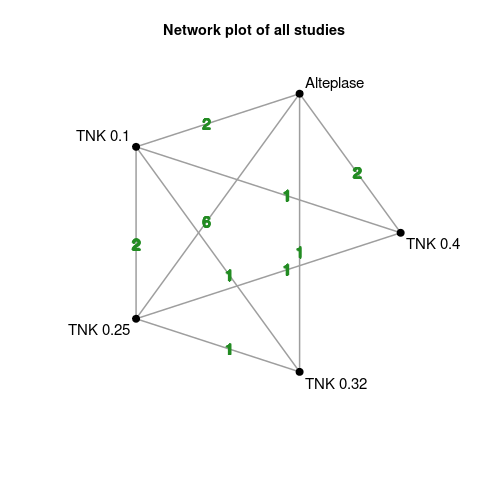


Figure S17: Network plot of any intracranial hemorrhage.


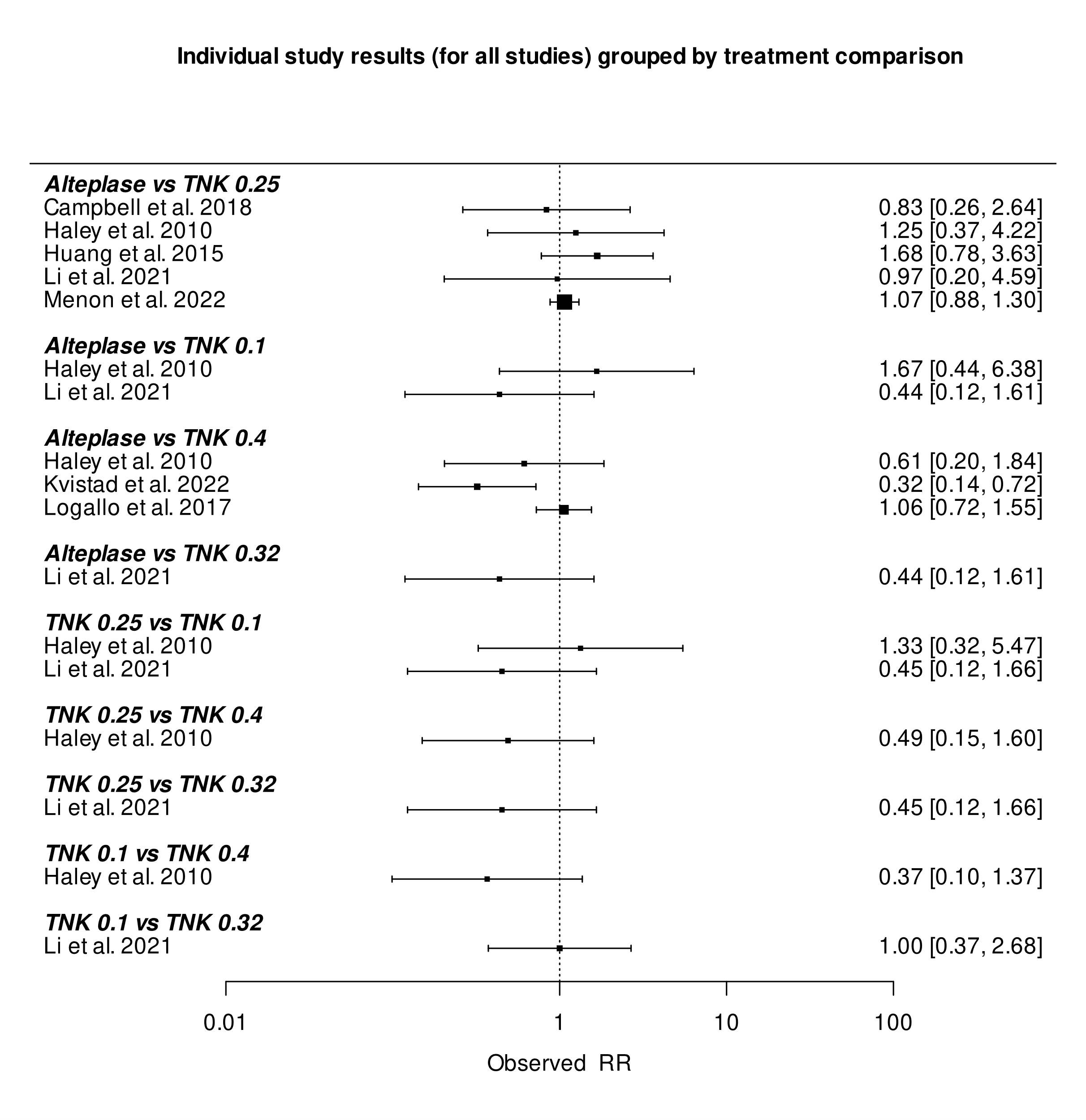


Figure S18: Forest plot of individual study results grouped by treatment component for any intracranial hemorrahge.


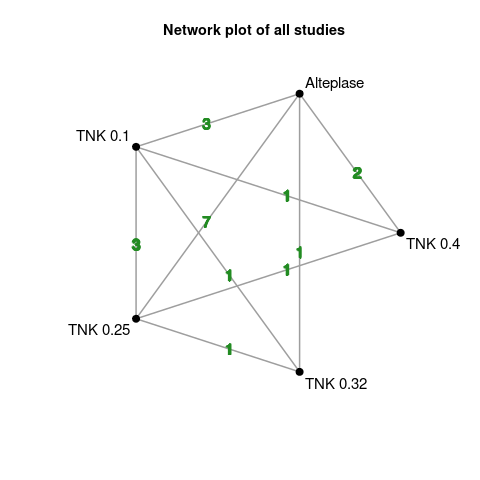


Figure S19: Network plot of symptomatic intracranial hemorrhage.


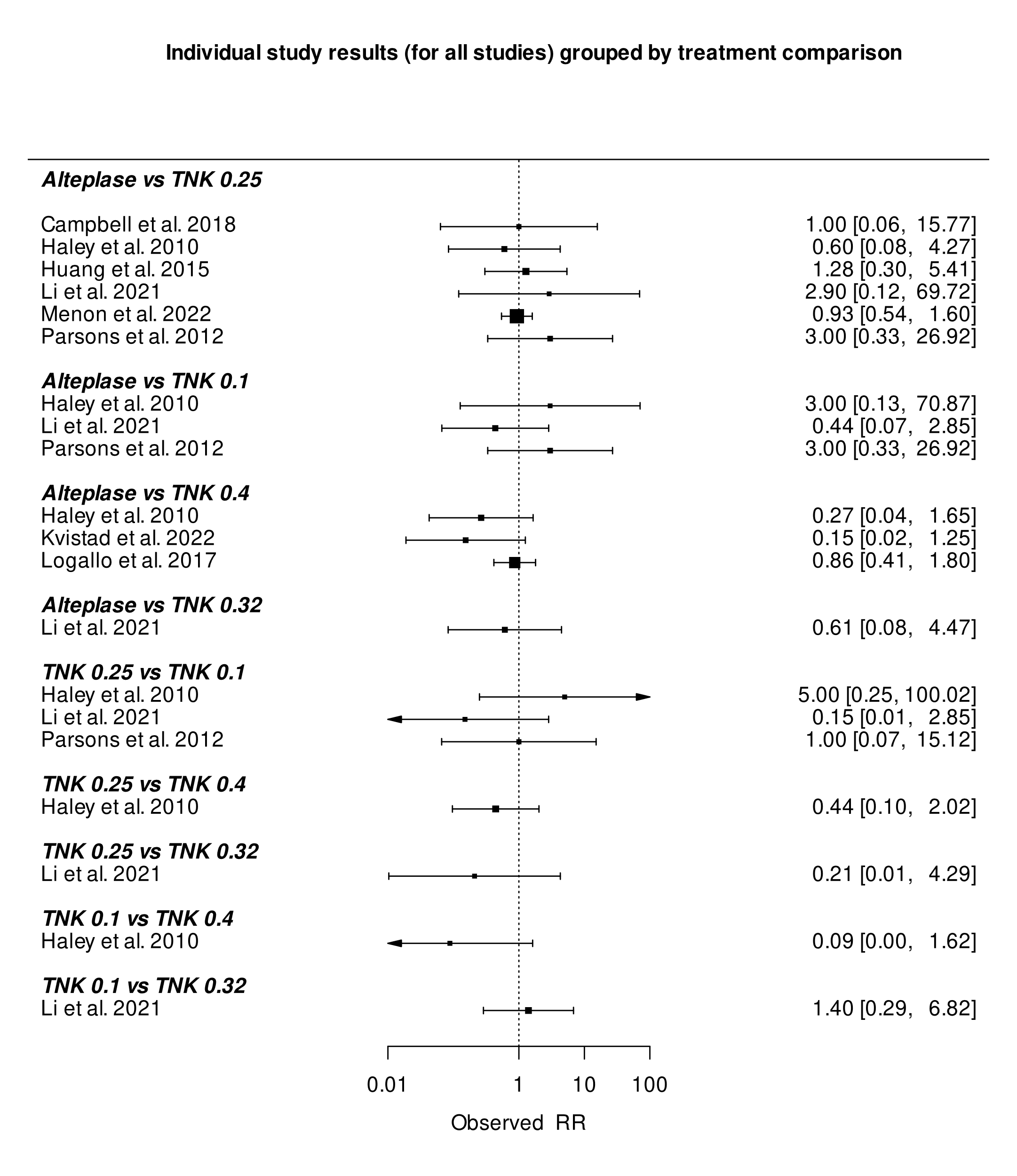


Figure S20: Forest plot of individual study results grouped by treatment component for symptomatic intracranial hemorrahge.


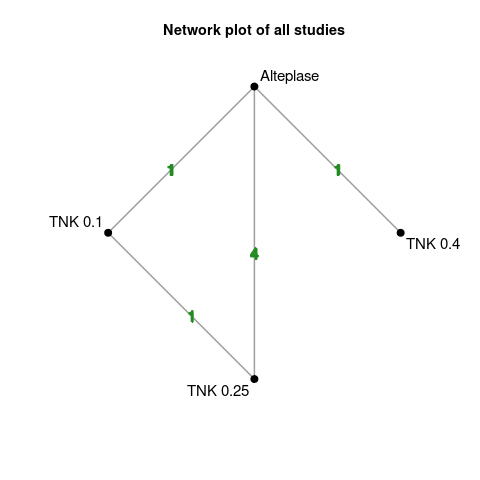


Figure S21: Network plot of any parenchymal hematoma.


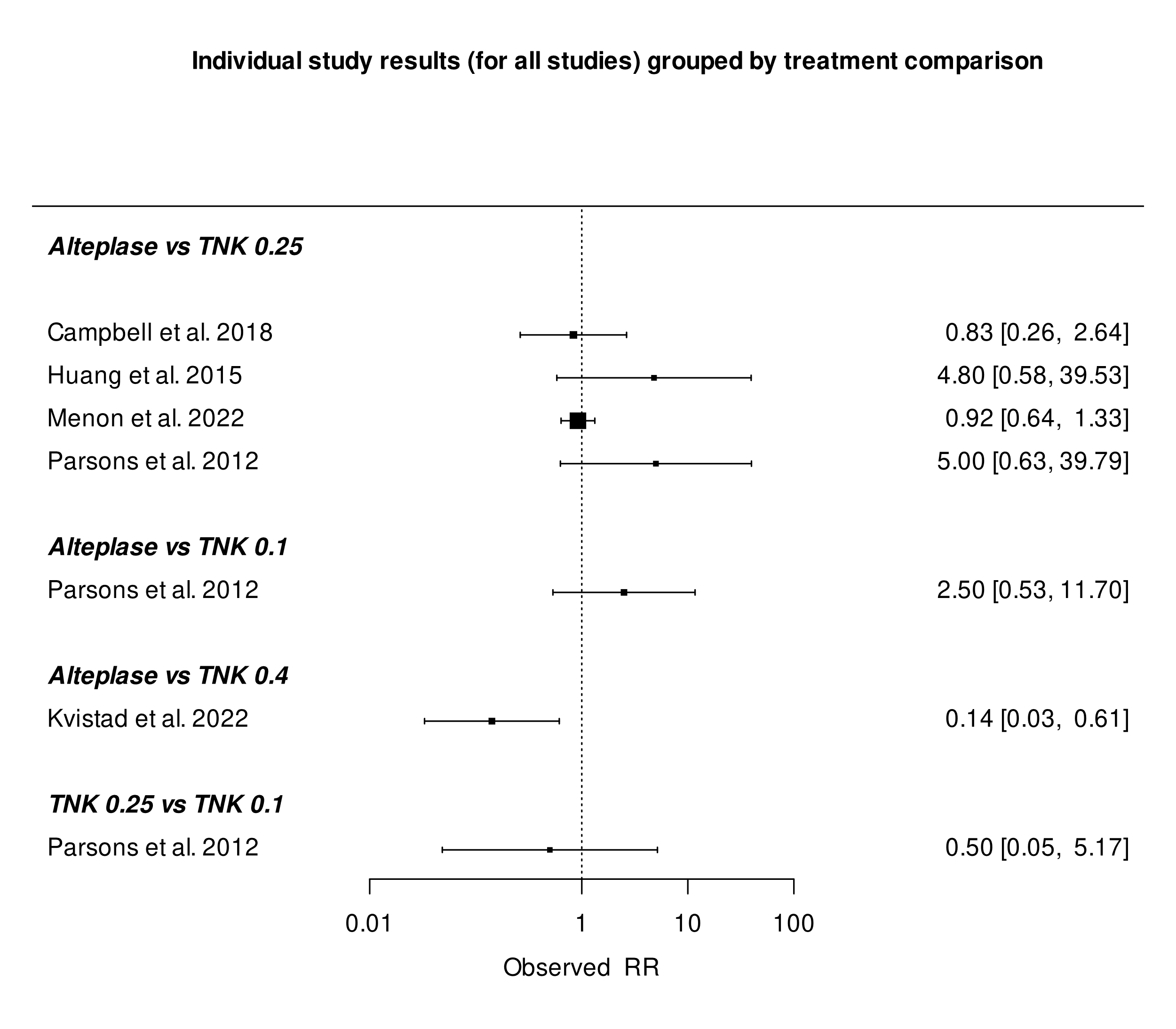


Figure S22: Forest plot of individual study results grouped by treatment component for any parenchymal hematoma.
